# Responsive Molecules for Organic Neuromorphic Devices: Harnessing Memory Diversification

**DOI:** 10.1002/adma.202418281

**Published:** 2025-03-26

**Authors:** Yusheng Chen, Bin Han, Marco Gobbi, Lili Hou, Paolo Samorì

**Affiliations:** ^1^ Université de Strasbourg CNRS ISIS 8 allée Gaspard Monge Strasbourg 67000 France; ^2^ Centro de Física de Materiales (CFM‐MPC) CSIC‐UPV/EHU Donostia‐San Sebastian 20018 Spain; ^3^ IKERBASQUE Basque Foundation for Science Bilbao 48009 Spain; ^4^ School of Precision Instruments and Optoelectronics Engineering Tianjin University Tianjin 300072 China

**Keywords:** artificial brain, memory diversification, neurotransmitter variation, organic neuromorphic devices, responsive molecules

## Abstract

In the brain, both the recording and decaying of memory information following external stimulus spikes are fundamental learning rules that determine human behaviors. The former is essential to acquire new knowledge and update the database, while the latter filters noise and autorefresh cache data to reduce energy consumption. To execute these functions, the brain relies on different neuromorphic transmitters possessing various memory kinetics, which can be classified as nonvolatile and volatile memory. Inspired by the human brain, nonvolatile and volatile memory electronic devices have been employed to realize artificial neural networks and spiking neural networks, respectively, which have emerged as essential tools in machine learning. Molecular switches, capable of responding to electrical, optical, electrochemical, and magnetic stimuli, display a disruptive potential for emulating information storage in memory devices. This Review highlights recent developments on responsive molecules, their interfacing with low‐dimensional nanostructures and nanomaterials, and their integration into electronic devices. By capitalizing on these concepts, a unique account of neurotransmitter‐transfer electronic devices based on responsive molecules with ad hoc memory kinetics is provided. Finally, future directions, challenges, and opportunities are discussed on the use of these devices to engineer more complex logic operations and computing functions at the hardware level.

## Graphical abstract

Responsive molecules are essential for organic in‐sensor computing devices. This Review highlights recent advances in thedesign, synthesis, and incorporation of electrically, optically, and magnetically responsive molecules in multifunctional synaptic perception devices endowedwith both nonvolatile and volatile memory diversification. By exploiting the multifunctional nature of molecular switches, complex logic operations can be accomplished, bringing molecule‐based neuromorphic computing closer to become a real technology.

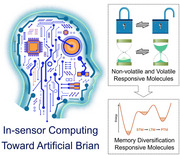

## 1 Introduction

During the past decade, the revolutionary advancement of artificial intelligence (AI) has imposed a paradigm shift in our society, driving significant progress in healthcare, environmental monitoring, finance, and entertainment. However, the growing demand for big data processing, has evidenced critical limitations in traditional computing systems based on von Neumann architecture, including high energy consumption and limited calculation speed, due to the transfer of data between the memory and processing units, which are separated. In contrast, the human brain operates with an ultralow energy consumption of 0.3 kilowatt hours (kWh) per day as it requires just 20 W of power, making it an exceptionally energy‐efficient system.^[^ ^1^ ^]^ Alongside its versatility, this unique feature has inspired scientists to consider neuromorphic computing architecture as a powerful alternative to the established von Neumann architecture.

The first investigation into the working mechanism of neurons was conducted by Lapicque in 1907.^[^ ^2^ ^]^ This pioneering study introduced the integrate‐and‐fire (IF) model, being an electrical circuit designed to emulate signal processing in biological neurons. In this model, neurons accumulate inputs and emit output spikes once a threshold is reached. Based on the IF model, a new computing paradigm known as artificial neural networks (ANNs) were first proposed in 1943, which mimic the structure of the brain by employing different layers of interconnected neurons to process data.^[^ ^3^ ^]^ While Lapicque's seminal work comprised the inherent decay feature of membrane potential of neuron, Stein mathematically refined the IF model in 1965, developing it into the leaky integrate‐and‐fire (LIF) model.^[^ ^4^ ^]^ Based on LIF model, spiking neural networks (SNNs) could more closely mimic biological neurons by generating output signals with dependence of the precise timing of discrete input spikes.^[^ ^5^ ^]^ Both ANNs and SNNs serve as foundational technologies that underpin modern advancements in AI. In these networks, electrical signals are integrated through circuitry until a threshold is reached, triggering the generation of an electrical output that is passed to the next node of the network. After processing through multiple hidden layers, the analytical result can be ultimately produced.

In a nutshell, neuromorphic devices combine in situ data processing with low power consumption, enabling the handling of complex tasks that involve parallel analysis of large datasets.^[^ ^6^, ^7^, ^8^, ^9^, ^10^ ^]^ Modern AI technology is seeking a breakthrough by integrating the concepts of sense digitalization and in‐sensor computing. Rather than transferring the data from edge‐sensory artificial organ to central data processing unit, in‐sensor computing offers the opportunity to process data directly within the sensor itself. This approach holds the promise for building efficient and intelligent systems. Such goal can be achieved through the development of multifunctional devices, which integrate the sensory, storage, and process functions into a single device.^[^ ^11^, ^12^, ^13^, ^14^ ^]^

**Figure 1:**
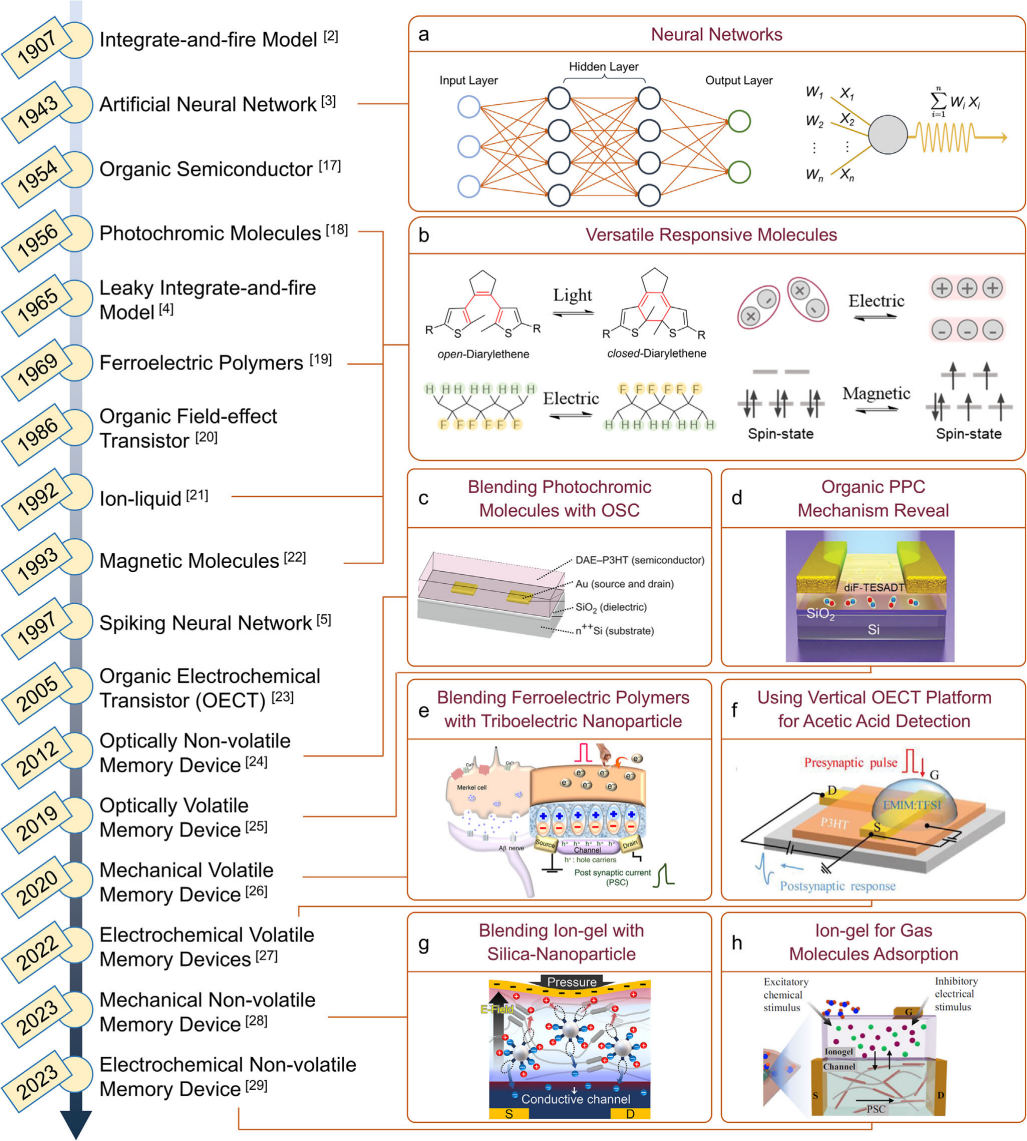
Timeline of milestone works on a) artificial neural network, b) responsive molecules and c–h) their integration in organic in‐sensor computing devices.^[^ ^2^, ^3^, ^4^, ^5^, ^17^, ^18^, ^19^, ^20^, ^21^, ^22^, ^23^, ^24^, ^25^, ^26^, ^27^, ^28^, ^29^ ^]^ (c) Reproduced with permission.^[^ ^24^ ^]^ Copyright 2012, Springer Nature. (d) Reproduced with permission.^[^ ^25^ ^]^ Copyright 2019, Wiley‐VCH. (e) Reproduced under the terms of the CC‐BY license.^[^ ^26^ ^]^ Copyright 2020, Springer Nature. (f) Reproduced with permission.^[^ ^27^ ^]^ Copyright 2022, Wiley‐VCH. (g) Reproduced under the terms of the CC‐BY license.^[^ ^28^ ^]^ Copyright 2023, AAAS. (h) Reproduced under the terms of the CC‐BY license.^[^ ^29^ ^]^ Copyright 2023, Springer Nature.

Advances in chemical synthesis made it possible to develop molecules that can be interconverted between two or more states when subjected to specific external stimuli of physical, chemical and biological nature.^[^ ^15^, ^16^ ^]^ These include, among others, photochromic and magnetoresponsive molecules, ferroelectric polymers and ion‐gels. The historical overview provided in **Figure** **1** highlights the milestones that have culminated in the recent development of organic in‐sensor computing device, demonstrating that the incorporation of these molecules into electronic devices is a viable strategy for the efficient integration of sensory and in‐memory calculation.^[^ ^2^, ^3^, ^4^, ^5^, ^17^, ^18^, ^19^, ^20^, ^21^, ^22^, ^23^, ^24^, ^25^, ^26^, ^27^, ^28^, ^29^ ^]^ Unlike inorganic materials, organic molecules hold the great advantages of enabling the fabrication of flexible, bendable, foldable, and stretchable electronic devices, which is the key for the efficient human–machine interfacing.^[^ ^30^, ^31^ ^]^

From the perspective of biological neuromorphic systems, the process of perception begins with the sensory organ responding to an external stimulus, resulting in temporary neuron spiking and the formation of short‐term memory (STM). Further rehearsal process of the external stimulus converts the STM into a long‐term memory (LTM) and a permanent‐term memory (PTM), which are known as early long‐term potentiation and late long‐term potentiation, respectively.^[^ ^32^, ^33^ ^]^ The STM and LTM rely on volatile processes that decay over time, such as ion flux and migration. Conversely, the PTM requires protein synthesis in neuron and even structural changing on dendritic spines. Extensive studies revealed that synaptic plasticity is dependent on the generation of the neurotransmitter receptors (i.e., *N*‐methyl‐d‐aspartate), which can be activated to form the ion channel (i.e., Ca^2+^). The STM and LTM do not require changes in transcription or translation, herein only lasting from seconds to hours. Instead, the PTM depends upon gene expression, activation of protein kinase, and the synthesis of memory‐related protein, therefore persistently influencing the synaptic connection weight and prolonging the memory to days and months, which are considered as learning processes.^[^ ^34^, ^35^, ^36^ ^]^ Biologists suggest that synaptic connections between neurons are not immutable but can be modified by learning and training, which indicates that both volatile memory and nonvolatile memory are essential for the memory and learning process in human brain.^[^ ^37^, ^38^, ^39^, ^40^ ^]^

During the last few years, a great research effort has been devoted to the design and synthesis of responsive molecules combining a nonvolatile and volatile nature for in‐sensor computing devices. So far molecules imparting either the characteristic of nonvolatility or volatility to memory devices have been used. Yet, in human brain, STM, LTM, and PTM operate cooperatively as crucial computing functions, to control the processes of training‐to‐learning and feedback training. In this Review, we summarize the most enlightening recent developments on the design of molecules capable of responding to electrochemical, photonic, and magnetic stimuli, with a special focus being dedicated to sensory stimulus storage. We discuss the integration of these responsive molecules into electronic devices, placing particular emphasis on device manufacturing technologies for empowering sensory devices with different memory retention times. More specifically, we highlight recently reported examples of engineering the transition from LTM to PTM. The integration of multiresponsiveness in specific optoelectronic devices allows the execution of complex logic algorithms, offering promising advancements toward the development of artificial brains. Finally, we discuss the challenges and opportunities for the 3D integration of flexible electronic synapse networks comprising responsive molecules.

## 2 Key Parameters for the Neuromorphic Devices

Neuromorphic devices are engineered to mimic the structure and functionality of biological synapses. These artificial synaptic devices operate by emulating the key characteristics of neurons and synapses, including dynamic adaptation, synaptic plasticity, and spiking behavior. Inspired by the complex connectivity between neurons in the brain, the neuromorphic devices are integrated as cross‐bar network architecture, enabling the efficient matrix calculations. In this section, we introduce the critical parameters of neuromorphic devices.

### 2.1 Dynamic Range

**Figure 2:**
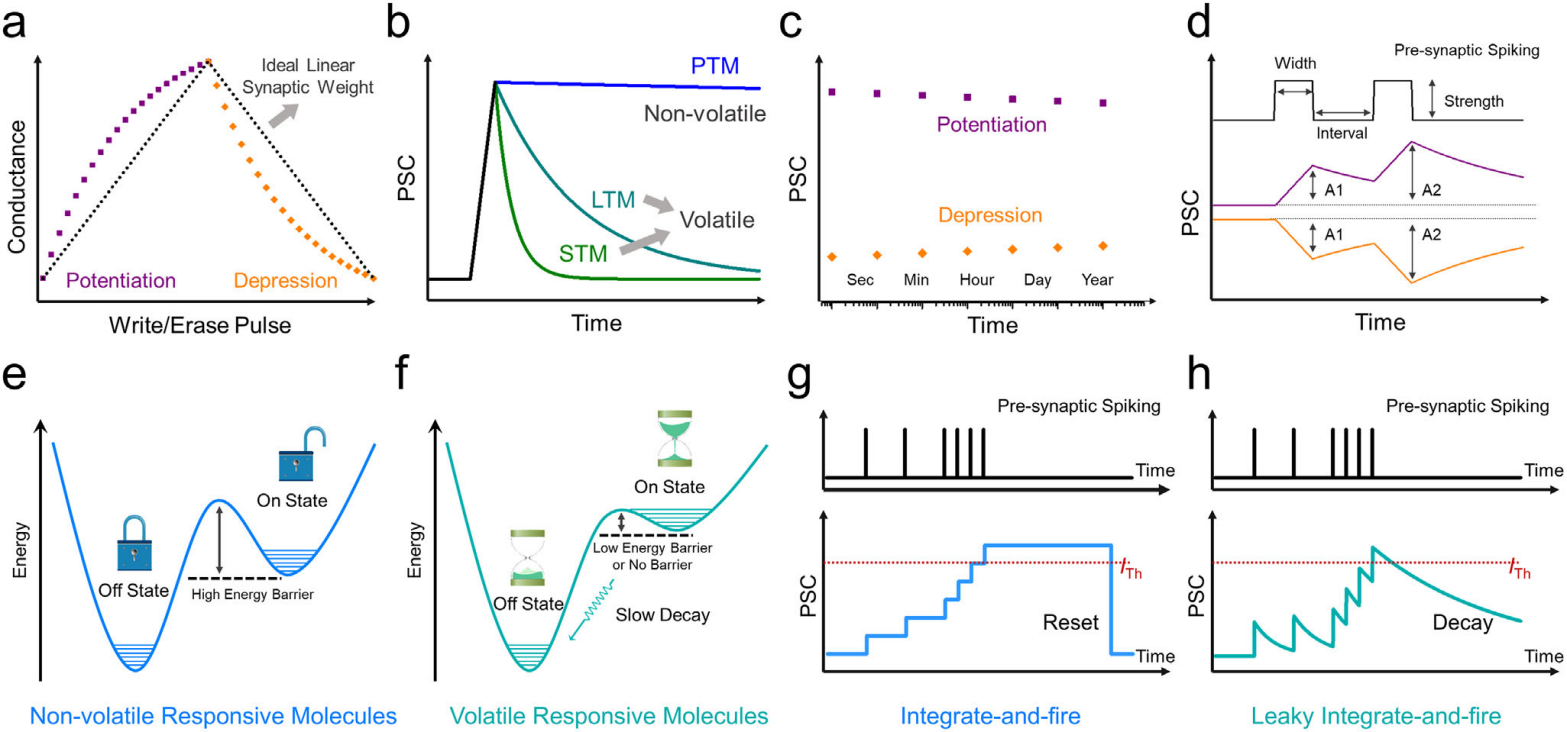
Schematic diagram of synaptic device characterization. a) Synaptic weight update scheme for potentiation and depression processes. b) Memory diversification behavior identified from PSC decay curves. c) Scatter plot measurement for the nonvolatile memory device. d) Spike‐time‐dependent plasticity. Thermodynamic energy diagram of e) nonvolatile responsive molecules and f) volatile responsive molecules. Scheme illustration of g) IF model and h) LIF model for the neuromorphic application.

In traditional computing systems, memory devices typically operate on a binary basis, recording data in bits that are labeled either as 0 or 1. This represents a major limitation for the processing of big data such as in image rendering. Conversely, neuromorphic computing overcomes this limitation by leveraging multilevel memory devices, which can store synaptic weights as continuous values, ruled by the conductance (*G*) of memory devices.^[^ ^41^, ^42^ ^]^ **Figure** **2**a portrays a typical synaptic weight update process during a series of write and erase pulses. The progressive increase/decrease of the device conductance reflects the potentiation/depression of the connection strength between the pre‐ and postartificial synapse. The ratio of maximum and minimum value of conductance can be defined as dynamic range of memory device, which is expressed as *G*_max_/*G*_min_. For example, during neural network operation, a device with *G*_max_/*G*_min_ amounting to 100 means that the minimum synaptic weight that can be represented is 0.01. Herein, by ensuring reliable reading in distinguishing synaptic weight and improving the calculation accuracy, the artificial synaptic device with a higher *G*_max_/*G*_min_, a lower cycle‐to‐cycle variation and a lower nonlinearity is desired.^[^ ^43^ ^]^

### 2.2 Memory Retention Time

The memory retention time is a crucial key performance indicator in a memory device, which can be extracted from the real‐time characterization of device after being subjected to the external stimulus (Figure 2b). In principle, the decay behavior of information storage follows the exponential decay equation

(1) $$I\left(t\right)=I_{0}\;e^{-t/\tau }+I_{\infty }$$

where the *I*_0_ is the initial current value, *I*_∞_ is the stable‐state current, and *τ* is the lifetime of memory storage. Unfortunately, guidelines and precise criteria to categorize memories and their diversification in electronic system are still missing. Nevertheless, in view of faster information processing speed of electronic device, the memory timescales of STM and LTM in electronic systems are expected being all shorter than those in biological neurons. Herein, we therefore propose a simplified timescale standardization for memory diversification in electronic neuromorphic systems, i.e., microseconds‐to‐seconds, seconds‐to‐hours, and hours‐to‐days for STM, LTM, and PTM, respectively. For electronic memory devices characterized by a nonvolatile nature, the scatter dot measurement can be utilized to evaluate the retention time (Figure 2c). Notably, the memory retention time of neuromorphic device can be prolonged after a series of setting training. Extensive research efforts have demonstrated that the successful STM‐to‐LTM transition can be achieved via stimulus rehearsal, which rules the enhanced postsynaptic current (PSC). However, the transition from LTM to PTM, i.e., from volatile to nonvolatile, is still a major challenge in the field of neuromorphic device, which will be discussed in Section 5.

Significantly, the chemical design of responsive molecules allows for the tailoring of their thermodynamic stability across different states, thereby enabling either nonvolatile or volatile data recording. On the one hand, nonvolatile responsive molecules can be toggled between two or more thermodynamically stable states (Figure 2e). The energy barrier between these states prevents backward switching at the initial state, making them ideal to emulate PTM. On the other hand, volatile responsive molecules exhibit unique kinetic decay properties, due to transition from a molecular excited state to the ground state or more generally from a thermally unstable state to a thermally stable state (Figure 2f). These properties are particularly important for creating dynamic memory systems. Volatile data storage with the property of STM to LTM transition enables the mimicking of artificial synaptic devices, wherein the forming of STM or LTM depends on timing and strength of stimulus spikes.

### 2.3 Paired‐Pulse Facilitation/Depression

Spike‐time‐dependent plasticity is an important biologically inspired learning rule, whose behavior can be simply evaluated through the analysis of paired‐pulse facilitation (PPF) index and paired‐pulse depression (PPD) index, indicating the enhancement or reduction of neurotransmitter release. As depicted in Figure 2d, by applying a pair of presynaptic pulses with various pulse width, strength, interval (frequency), the PPF/PPD index is defined as the increasing/decreasing percentage of second PSC value (*A*_2_) compared with first PSC value (*A*_1_)

(2) $$\mathrm{PPF}/\mathrm{PPD}=\left(A_{2}-A_{1}\right)\;÷A_{1}\times 100\%$$

The PPF/PPD index of nonvolatile memory device is solely influenced by the spike width and strength, as it is characterized by spike‐width‐dependent plasticity and spike‐strength‐dependent plasticity. However, in volatile memory devices, PPF/PPD index gradually decreases with the increasing time interval (Δ*t*) between the pair of pulses, because of the decay of the synaptic weight. Herein, the synaptic plasticity of volatile memory devices is further influenced by the spike‐interval and spike‐rate, which are known as spike‐interval‐dependence plasticity and spike‐ratio‐dependence plasticity.

### 2.4 Neural Networks Calculation

Neural networks calculation is implemented by processing raw data through multiple hidden layers with interconnected neurons. As illustrated in the top‐right panel of Figure 1, the presynaptic input is represented as W, while the connection strength of neurons is denoted as X. Each neuron computes the synaptic weighted sum from its inputs spiking (expressed as ∑W*_i_* X*_i_*), followed by the application of an activation function to decide whether to pass the result to the next layer. This process is carried out iteratively until the final calculation result is obtained. During the training process, the connection strength is adjusted, enabling the improving of calculation accuracy.

Nonvolatile memory devices enable persistent data storage without requiring of continuous power stimulus input, thus allowing their adaptive application in ANNs. Vector‐matrix multiplication can be performed in the layer of nonvolatile memory device using cross‐bar array architectures, which relies on the Ohm's law and Kirchhoff's current law. The IF model offers a simple solution to implement ANNs (Figure 2g). Once the input stimulus is applied, the neuron stores the incoming presynaptic spiking over time, leading to an increase of PSC of the neuron. When the PSC reaches a predefined threshold (*I*_Th_), the neuron “fires,” emitting a spike to the next neuron which can be conceived like a binary “1”. According to IF model, after a neuron fires, a resetting instruction is necessary for bringing the PSC back to the ground state, denoted as “0”. This resetting instruction makes the neuron ready for subsequent operation, maintaining the fidelity and accuracy of the processing system.^[^ ^44^ ^]^

SNNs are another efficient neuromorphic computing models, which take the advantages of volatile memory device, to mimic brain activity by encoding information as temporal spikes. The LIF model is utilized to interpret the time‐dependence behavior of volatile memory (Figure 2h). In analogy to IF model, the operation of LIF model integrates the input spiking, reflecting as the PSC increases. Yet, the LIF model introduces one additional “leaky” term, accounting for the natural decay of PSC over time. This feature obviates the need for explicit resetting instructions once the “fire” is finished, due to current of neurons naturally reverting to the ground state over time, which makes LIF model more efficient. However, based on the LIF's time‐depended plasticity, SNNs require complicated training algorithms and well‐established datasets, restricting its application to event‐driven analyses.

In both IF model and LIF models, the “fires” process is executed by a current comparator that is connected on the in‐memory computing devices. The neuron fires only when the PSC exceeds the threshold set by the current comparator, completing the “fires” process. Notably, light‐emission function can be integrated into the in‐memory computing device to accomplish the fire process, thereby contributing to a built‐in threshold.^[^ ^45^, ^46^, ^47^, ^48^ ^]^ In the specific case of multilevel memory light‐emitting devices, the light‐emitting materials can be only driven when the PSC exceeds the threshold current (or turn‐on current), leading to the inherent determination of “fire” process. By and large, both ANNs and SNNs are aimed at emulating the neural architecture and functionality of the human brain, by simulating the way neurons process and transmit information through electrical potentials.

### 2.5 Energy Consumption

Energy consumption is a paramount aspect for neuromorphic computing applied in energy‐constrained environments to process big data. In the context of single synaptic spiking event, energy is consumed during both stimulus programming and PSC read‐out processes, which are defined as presynaptic energy consumption (*E*_Pre_) and postsynaptic energy consumption (*E*_Post_), respectively. The *E*_Post_ is simply calculated by the postsynaptic voltage (*V*_Post_), PSC, and postsynaptic period (*t*), with the following equation

(3) $$E_{\mathrm{Post}}=V_{\mathrm{Post}}\;\times \mathrm{PSC}\times t$$

Conversely, the complex *E*_Pre_ analysis is partially ignored in current research foci, because of the different evaluation methods for various in‐sensory synaptic devices. For example, in photosensing synaptic device, the *E*_Pre_ is calculated by estimating the power of input light irradiation (*P*_Light_)

(4) $$E_{\mathrm{Pre}}=P_{\mathrm{Light}}\;\times t$$

In the mechanical‐sensing synaptic device, the *E*_Pre_ is calculated by presynaptic voltage (*V*_Pre_) applied on mechanical sensor or by force (*F*) and distance (*D*) (self‐power sensory device issue)

(5) $$E_{\mathrm{Pre}}=V_{\mathrm{Pre}}\;\times I_{\mathrm{Pre}}\times t=F\times D$$

However, in biomolecular‐ and gas‐sensing synaptic devices, reliable methods to evaluate the energy consumption of the input stimulus are complicated. Overall, according to Equations (3)–(5), the reduction of the energy consumption of neuromorphic devices can be achieved by increasing the conductance and enhancing the responsiveness of devices.

## 3 Responsive Molecules with Memory Recordability

Stimuli‐responsive molecules can act as smart materials, which are capable to respond to various external physical (heat, light, electric/magnetic fields, humidity, pressure) and chemical inputs by changing their states. Leveraging such versatility of responsive molecules, advanced technologies including molecular motors, artificial muscles, soft robots have been developed.^[^ ^49^, ^50^ ^]^ To integrate sensory and memory functions into electronic devices, responsive molecules powered by light, electrical, redox or magnetic stimuli appear particularly adapt.

### 3.1 Optically Responsive Molecules

The use of light as a stimulus has some unique advantages: it is noninvasive, it does not require a physical contact, it can be tuned across a wide range of intensities and wavelengths, and it can be remote controlled with high spatial‐temporal resolution. Conjugated molecules with bandgap ranging between 1.2 and 3.0 eV can absorb UV–vis light, yielding the electron's excitation from the π (or *n*) orbital to the π* orbital. However, the excited electron decays quickly due to the absence of an energy barrier. To endow a memory function, a storage energy level should be introduced to retain the electron in a given state for a certain period of time. The photogenerated carriers (electrons and holes) enhance the conductivity or mobility of the electronic devices. The ability of modulating current flow in response to light can be used to mimic brain neural processes such as learning (strengthening synaptic connections) and the transition from STM to LTM in artificial synapses (see Section 4.1.2)

On the other hand, photochromic molecular switches, based on reversible photochemical reactions, such as photocyclization/photocycloreversion, *trans*/*cis* photoisomerization, can reversibly change their molecular structures when exposed to light at specific wavelengths.^[^ ^51^, ^52^, ^53^, ^54^, ^55^ ^]^ The light induced reversible transformations involve changes of geometry, charge distribution, or bond configurations, which in turn lead to modifications in their physical properties, such as color, energies in the highest occupied molecular orbital (HOMO) and lowest unoccupied molecular orbital (LUMO), electron affinity, or conductivity. When such molecular photoswitches are integrated in optoelectronic devices, it is possible to exploit their photoresponsive nature to tune the device's electrical and/or optical properties,^[^ ^56^, ^57^, ^58^ ^]^ by interconverting them between different states, similarly to the synapses switching between firing and resting states. When toggled upon irradiation at specific wavelengths, the light‐responsive molecules integrated in the devices can retain their state without requiring of continuous energy input, in analogy to a nonvolatile memory behavior, to enable the emulation of PTM. This is critical for creating energy‐efficient synaptic devices that retain information even when the power is removed (see Section 4.1.1).

**Figure 3:**
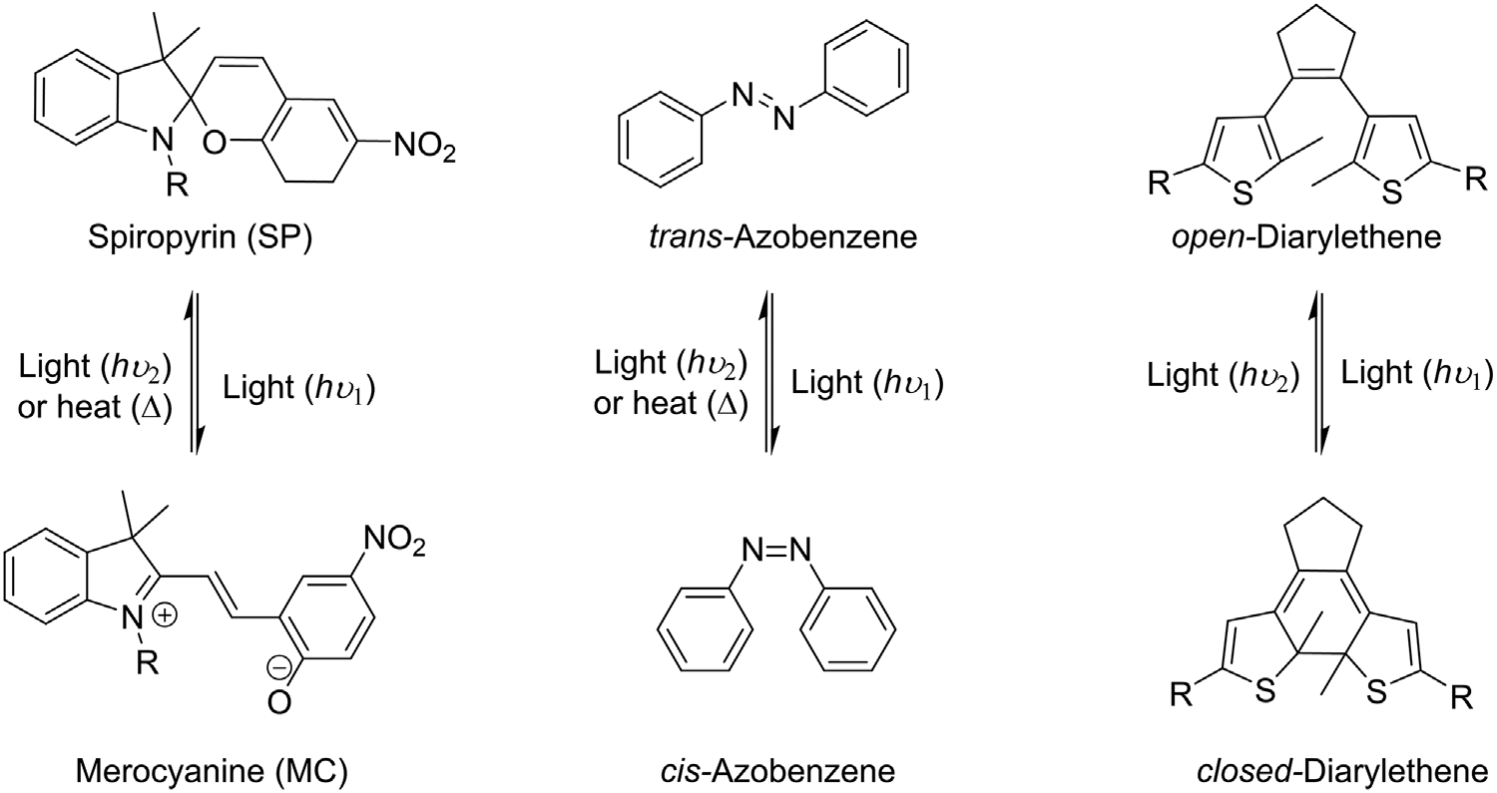
Chemical structure of the archetypical photochromic molecules and their isomerization processes.

The common molecular photoswitches employed to fabricate optically responsive artificial synapse are spiropyran/merocyanine (SP/MC),^[^ ^59^, ^60^ ^]^ azobenzene (Azo),^[^ ^61^, ^62^ ^]^ and diarylethene (DAE)^[^ ^63^, ^64^ ^]^ whose chemical structures are portrayed in **Figure** **3**.

i) SP exists in a closed‐ring structure, where the indoline and chromene units are connected via a spiro carbon atom. SP exhibits nonpolar, colorless properties with limited electron delocalization. Upon exposure to UV light, the bond cleavage at the spiro carbon induces the formation of open‐ring MC zwitterionic structure. The MC form is highly conjugated, colored, more polar and it possesses higher conductivities. The MC form is generally thermodynamically unstable, as it can revert back to the SP isomer upon heating in the dark or under visible light irradiation. The reversible changes in transmittance when exposed to UV and visible light irradiation is analogue to the excitatory postsynaptic intensity and inhibitory postsynaptic intensity in an all‐optical artificial synapse.^[^ ^65^, ^66^ ^]^
ii) Azobenzene undergoes reversible photoisomerization between the *trans* and *cis* forms. The *trans* form has a linear structure with the two phenyl rings positioned on opposite sides of the central N═N bond. This form is the more stable (i.e., thermodynamically stable) and it is usually nonpolar in view of its low dipole moment. When exposed to UV light, upon rotation or inversion the two phenyl rings bend on the same side, yielding the more compacted *cis* form. The less stable (i.e., metastable) and more polar *cis* form, due to its higher dipole moment, can revert back to the *trans* form either thermally over time or upon exposure to visible light. By controlling molecular geometry and polarity of azobenzene with light, one can adjust the electronic properties of the devices changes thereby mimicking synaptic plasticity and memory functions.^[^ ^67^, ^68^, ^69^, ^70^ ^]^
iii) DAE undergoes reversible photoisomerization between the ring‐open and ring‐closed forms. The nonconjugated ring‐open form is colorless with a larger energy bandgap. Upon exposure to UV light, the molecule undergoes isomerization yielding the ring‐closed structure. The ring‐closed form is highly conjugated, colored, and it exhibits a smaller bandgap. The reverse isomerization process only occurs upon irradiation with visible light. In the case of DAE, both open and closed isomers are thermodynamically stable. In view of the unique properties of the open and closed states of DAE, its light‐induced switching also allows to control charge trapping and/or conductivity and synaptic weight modulation, like synaptic plasticity in biological systems.^[^ ^71^, ^72^ ^]^

Noticeably, both SP and Azo photoswitches are generally employed as volatile memory, as their thermodynamically unstable configurations can revert to the original state over time at room temperature. The timescale of such back‐isomerization is of seconds‐to‐minutes for MC to SP, and hours for *cis*‐Azo to *trans*‐Azo. Unlike SP/MC and Azo photoswitches, the thermodynamic stability of both DAE forms enables information to be stored in a nonvolatile manner without continuous energy input, thereby allowing the retention of synaptic states (memory) after light stimulation.

The fine tuning of the properties of these photochromic molecules can be achieved via their ad hoc chemical functionalization. For example, the functionalization of SP and Azo photoswitches with electron‐donating or electron‐withdrawing groups makes it possible to adjust the energy barrier of the thermal relaxation process to achieve desired memory time.^[^ ^59^, ^62^, ^73^ ^]^ The short relaxation time of SP or Azo photoswitches existing in solutions can be significantly increased by embedding these molecules in a viscous media such as a solid or polymer matrices, reaching spontaneous back isomerization timescales of hours or even days,^[^ ^59^, ^74^ ^]^ thus emulating LTM or PTM. DAE photoswitches are commonly interfaced with semiconducting materials, such as organic polymers and 2D materials,^[^ ^24^, ^75^, ^76^ ^]^ to create nonvolatile memory. Recently, Hou et al. combined DAEs with CdS quantum dots (QDs) to achieve all‐visible‐light driven nonvolatile memory both in the solution and solid state.^[^ ^77^ ^]^ In the design of a bicomponent system comprised of PbS QDs and carboxylate DAE photoswitches,^[^ ^78^ ^]^ the photoluminescence (PL) in the NIR region was herein modulated in a nonvolatile fashion via the photoisomerization of DAEs due to efficient triplet energy transfer.

### 3.2 Electric‐Field‐Responsive Molecular Systems

Molecules responding to electrical stimuli play an important role in neuromorphic computing devices, which can interact with various sensing devices. In three‐terminal architectures, the drain–source current can be modulated using an external electrical input applied to the gate electrode. For neuromorphic applications, materials such as ionic liquids and ferroelectric polymers are frequently utilized as gate dielectrics to effectively modulate the input signals, offering the advantage of tuneable electrical and ionic responses.^[^ ^79^, ^80^, ^81^, ^82^ ^]^

**Figure 4:**
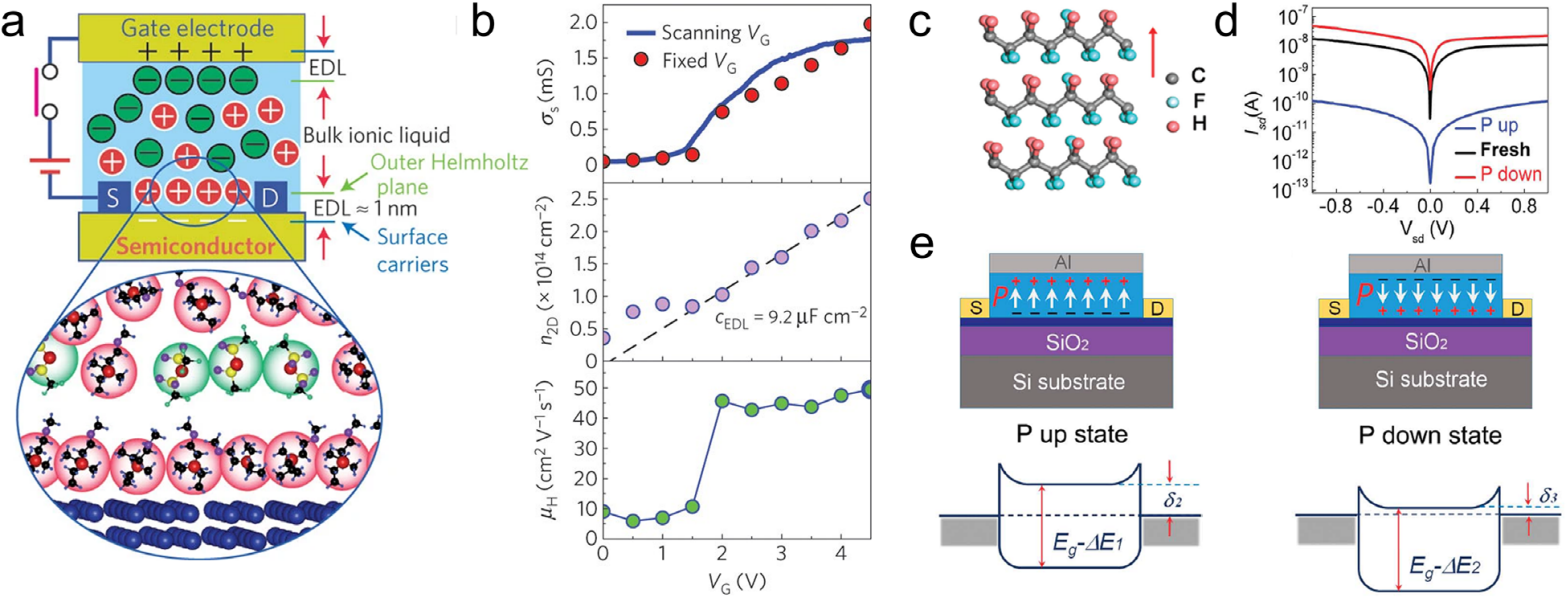
Electric‐field‐responsive molecular systems. a) Ionic liquids can be used as gate dielectric, as shown in this example for the van der Waals material ZrNCl. The application of a voltage across the liquid generates an electrical double layer, which introduces an ultrahigh electric field in the channel. b) The conductivity, charge carrier density and mobility were extracted for different values of the gate voltage. A capacitance of 9.2 µF cm^−2^ was estimated for the electrical double layer. (a,b) Reproduced with permission.^[^ ^87^ ^]^ Copyright 2010, Springer Nature. c) Chemical structure of the ferroelectric polymer P(VDF‐TrFE). d) Source–drain current measured in a MoS_2_ channel for opposite orientations of the polarization of P(VDF‐TrFE). e) Energy band diagram accounting for the different currents measured in (d). (c–e) Reproduced with permission.^[^ ^100^ ^]^ Copyright 2015, Wiley‐VCH.

i) Ionic liquids or electrolyte solutions are typically employed to realize electric double layer transistors.^[^ ^83^, ^84^, ^85^ ^]^ These devices consist of a (semi)conducting channel interfaced with the electrolyte, which is in also placed in contact with a metal electrode, serving as the external electrical input (gate). When a voltage is applied, an electric field is generated promoting the ions movement in the electrolyte toward the surface of the conducting channel, forming two electrical double layers, one at the gate electrode and one at the channel. If a positive gate bias is applied, anions accumulate at the surface of the gate electrode, while cations form the second ionic layer in the double layer. At the conducting channel, the opposite process occurs, with cations forming the first layer and anions the second.^[^ ^86^ ^]^ These electronic double layers generate an extremely high electric field, up to 10 MV cm^−1^.^[^ ^87^, ^88^ ^]^ This can be understood by considering that the externally applied voltage (usually ≈1 V) drops across the very short distance of the double layer (≈1 nm). Therefore, the electronic double layer acts as a dielectric with ultrahigh capacitance (above 10 µF cm^−2^),^[^ ^89^, ^90^ ^]^ enabling significant charge carrier accumulation in the conducting channel, with densities surpassing 10^14^ charges cm^−2^.^[^ ^87^, ^89^, ^90^, ^91^ ^]^ This density is one order of magnitude higher than what can be achieved with conventional dielectric as SiO_2_. Notably, these electric fields are achieved with relatively low voltage applied at the gate, being limited by the electrochemical stability window of the electrolyte (typically ±3 V). For example, in the case of **Figure** **4**a,b, the ionic liquid *N*,*N*‐diethyl‐*N*‐(2‐methoxyethyl)‐*N*‐methylammonium bis (trifluoromethylsulphonyl) imide (DEME‐TFSI) was used as gate dielectric to modulate the physical properties of the van der Waals material ZrNCl.^[^ ^87^ ^]^ The authors extracted the conductivity, the charge carrier density and the charge carrier mobility for the system at different applied voltages across the ionic gate. Based on the induced charge carrier density, the capacitance of the electron double layer was estimated as *C*_EDL_ = 9.2 µF cm^−2^, being over three orders of magnitude higher than the capacitance of a 100 nm thick SiO_2_ layer. Such high electric fields induced superconductivity at the surface of the ZrNCl semiconducting channel. Similarly, ionic liquid transistors have been used to explore gate‐induced superconductivity in different materials,^[^ ^89^, ^92^ ^]^ as well as magnetism^[^ ^93^, ^94^ ^]^ and phase changes.^[^ ^95^ ^]^ Remarkably, the electrical double layer generated by the ionic migration is volatile. The kinetics of the system are determined by the diffusion of the ions in the liquid medium, reflecting the movement of ions in neurons and synapses, thereby offering interesting possibility for SNNs (see Section 4.3). However, in some cases, the ultrahigh electric fields induce a permanent chemical or structural modification in the channel material, being of interest for nonvolatile memories.

ii) Ferroelectric polymers possess a spontaneous polarization which can be switched upon applying an external voltage.^[^ ^96^ ^]^ The prototypical ferroelectric polymer is polyvinylidene fluoride (PVDF), composed of (CH_2_–CF_2_) monomers. In PVDF, a highly polar structural phase can be stabilized with adjacent chains displaying an all‐*trans* conformation.^[^ ^97^ ^]^ In this phase, the strong dipoles of each monomer point in the same direction, resulting in a macroscopic polarization, which can be reversed upon applying a voltage. Importantly, the ferroelectric polarization is nonvolatile, as it remains even when the voltage is removed. Poly(vinylidene fluoride‐trifluoroethylene), P(VDF‐TrFE), a copolymer of vinylidene fluoride (VDF) and trifluoroethylene (TrFE), is often employed instead of PVDF, as its ferroelectric phase can be obtain more easily from solution.^[^ ^98^, ^99^ ^]^ Even in this phase, the electrical dipoles of the monomers are oriented along the same direction, providing an electric‐field switchable macroscopic polarization (Figure 4c). Figure 4d,e displays an example of how the ferroelectric properties of P(VDF‐TrFE) can be exploited in field‐effect transistors (FETs). The authors fabricated an FET based on a MoS_2_ channel and a P(VDF‐TrFE) gate dielectric. In this device, the drain–source current depended strongly on the polarization state of P(VDF‐TrFE). Specifically, a low current was recorded with an upward polarization, whereas the downward polarization resulted in a three‐orders‐of‐magnitude higher current. This effect was attributed to how the electric field from the P(VDF‐TrFE) polarization affects the band structure of MoS₂.^[^ ^100^ ^]^ In the upward‐polarization state, the current injection from the metal electrode into MoS_2_ encounters a large energy barrier. In the downward state, the conduction band of MoS_2_ is lowered in energy, reducing the energy barrier and favoring the charge injection. Importantly, these data were recorded without applying an external gate voltage when operating the device, as the polarization state of the ferroelectric polymer was preset in the desired direction by applying an appropriate voltage prior to the experiment. This result provides unambiguous evidence of the nonvolatile nature of the electric field generated by the ferroelectric polymer. Interestingly, P(VDF‐TrFE) is not only ferroelectric, but also piezoelectric,^[^ ^101^ ^]^ offering solutions for applications as actuators and pressure sensors, which are highly relevant in neuromorphic computing (see Section 4.2.2).

### 3.3 Magnetic‐Field‐Responsive Molecular Systems

Humans do not have the ability to sense magnetic stimuli, in stark contrast to some animals such as migrating birds. Developing magnetic perception for AI would offer interesting prospects for advanced location recognition and navigation systems technologies. In some molecular systems, the application of a magnetic field triggers a response, which can be converted to an electrical output under appropriate conditions.^[^ ^102^ ^]^ These magnetic field‐responsive molecules can be integrated into functional devices, providing an additional perception to generate electrical signals with tuneable kinetics in response to magnetic fields. These molecules represent a potential alternative approach to neuromorphic computing, offering capabilities beyond those of the human brain.

Several paramagnetic molecules, including stable radicals and metalorganic compounds, possess unpaired spins.^[^ ^103^, ^104^ ^]^ Although these molecules are sometimes referred to as “magnetic,” their spin state can be not stabilized or controlled on demand. In contrast, other compounds allow for a controllable manipulation of the spin configuration. Here, we briefly present three classes of molecular systems characterized by spin switching: spin crossover molecules, single‐molecule magnets, and magnetic metal–organic frameworks (MOFs).

**Figure 5:**
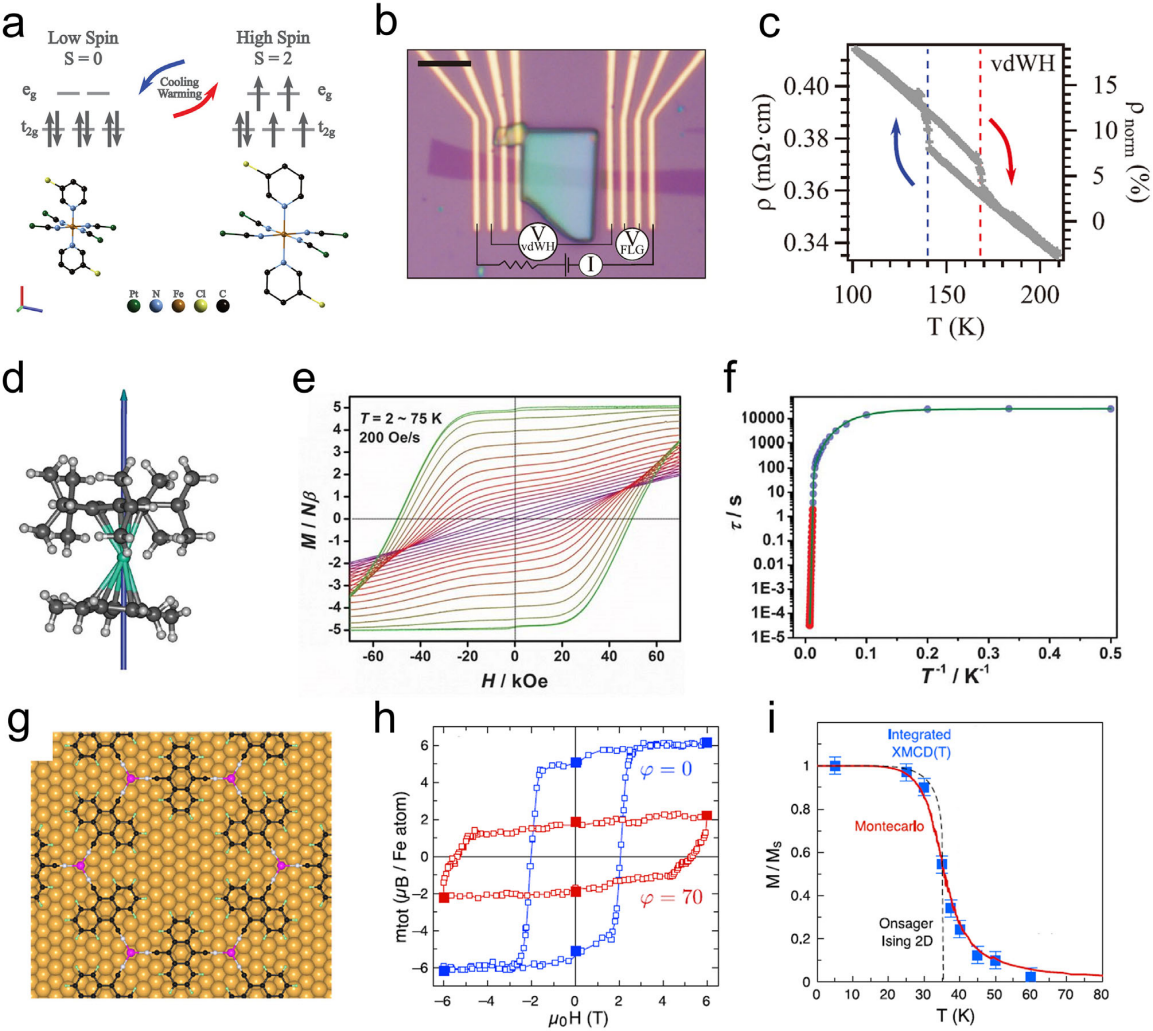
Magnetic‐field‐responsive molecular systems. a) Energy levels of the low spin state and high spin state of a Fe(II) spin crossover molecule. The difference in molecular volume is exaggerated for clarity. b) Device employed to electrically readout the switching of a spin crossover crystals. c) Temperature dependence of the device resistivity in the thermal hysteresis window. (a–c) Reproduced with permission.^[^ ^111^ ^]^ Copyright 2022, Wiley‐VCH. d) Chemical structure of the single molecule magnet showing magnetic hysteresis above 80 K. e) Magnetic hysteresis measured at different temperatures and f) variation of the spin relaxation time for the compound in (d). (d–f) Reproduced with permission.^[^ ^117^ ^]^ Copyright 2018, AAAS. g) Ferromagnetic metal–organic coordination network on gold, showing Fe atoms (pink) connected through organic ligand. h) Magnetic hysteresis measured with a field applied out of plane (blue) and with an angle of 70° with respect to the normal direction. i) Temperature dependence of the magnetization, showing a Curie temperature of 35 K. (g–i) Reproduced under the terms of the CC‐BY license.^[^ ^118^ ^]^ Copyright 2024, Springer Nature.

Spin crossover molecules are metallorganic compounds that can switch between two distinct spin states—low‐spin and high‐spin—in response to external stimuli such as temperature, pressure and light.^[^ ^105^, ^106^ ^]^ The most common class of spin crossover molecules are Fe(II) complexes with octahedral geometry (**Figure** **5**a), where the metal center can be switched between a diamagnetic low‐spin (*S* = 0) and a paramagnetic high‐spin (*S* = 2) state.^[^ ^107^ ^]^ Typically, temperature is used as a trigger for the switching process, leading to a nearly complete low‐to‐high spin transition. Notably, for single crystals composed of orderly arranged spin crossover complexes, this transition is often sharp due to cooperative effects and it exhibits a thermal hysteresis. Within this hysteresis loop, two stable states can be repeatedly addressed by thermal cycling. This bistability, which often persists at room temperature, makes spin crossover compounds promising candidates for PTM. The cooperative effects driving the sharp transition are observed even in crystals with lateral sizes in the nanometer range, which can be coated with shell molecules and utilized as spin‐switch nanoparticles.^[^ ^108^ ^]^

The transition between spin states is accompanied by a notable difference not only in the magnetic properties, but also in their color, conductance, dielectric constant, and molecular volume. In particular, the volume change has been used in various works to generate strain and trigger an optical or electrical output.^[^ ^109^, ^110^ ^]^ An example is shown in Figure 5b,c.^[^ ^111^ ^]^ In this case, the Coronado's group placed few‐layer graphene on top of a crystal of spin crossover molecules and measured its resistance as a function of temperature. A thermal hysteresis in the graphene resistance is recorded, which mimics the transitions of the spin crossover crystal. This phenomenon occurs because the resistance of graphene is particularly sensitive to strain, thereby enabling electrical readout of the spin crossover transition. While thermal energy is the most common trigger for spin crossover, other stimuli such as light irradiation and pressure can also activate the transition. Additionally, recent works have shown how the spin crossover phenomenon can be combined to magnetoelectric coupling and ferroelectricity, hence providing an additional electric‐field tunabilty.^[^ ^112^, ^113^ ^]^ Notably, despite the magnetic nature of spin crossover complexes, magnetic fields are relatively inefficient at triggering the transition. Consequently, these molecules should be more accurately considered thermally or optically responsive compounds.

Single molecule magnet is an example of a molecular system where the spin state can be modified using a magnetic field.^[^ ^114^ ^]^ In conventional paramagnetic molecules, the unpaired spin flips randomly and rapidly between different directions. In contrast, in single molecule magnets the spin remains locked in a particular direction below a certain blocking temperature due to a built‐in magnetic anisotropy.^[^ ^115^ ^]^ Under these conditions, the molecular spin can be controllably reversed through an external magnetic field, giving rise to a magnetic hysteresis. For decades, single‐molecule magnets exhibited low blocking temperatures, typically below 20 K, but more recent works have identified compounds displaying magnetic hysteresis above 60 K.^[^ ^116^ ^]^ Figure 5d shows the chemical structure of one of these compounds, which displays a finite hysteresis also at 75 K (Figure 5e).^[^ ^117^ ^]^ Correspondingly, the spin relaxation time, or the average time between random spin flip events, varies several orders of magnitude at different temperatures (Figure 5f). In this regard, the temperature can be employed to finely tune the kinetics of the system, providing LTM behavior at low temperature and a STM at higher temperature. While this tuneability would be ideal for the neuromorphic computing approach proposed here, it is worth noting that the electrical readout of the molecular spin state is far from being straightforward, and it has only been accessed in complex and not‐scalable single‐molecule devices.

Finally, we highlight that the magnetic hysteresis of single molecule magnets resembles that of conventional ferromagnetic system, yet they are fundamentally different. While individual single molecule magnets are characterized by hysteretic behavior of isolated spins, ferromagnetic systems are characterized by long range magnetic ordering. Notably, this long‐range ferromagnetic order has been recently reported in 2D MOFs self‐assembled on an Au surface, in which Fe atoms are coordinated through organic linkers (Figure 5g).^[^ ^118^ ^]^ The hysteresis loop measured for this system, shown in Figure 5h, displays a remarkably high coercive field of 2T and a strong out‐of‐plane magnetic anisotropy. The effect vanished above a Curie temperature of 35 K (Figure 5i). Intriguingly, this work opens the way for the study of different combinations of substrate, magnetic ions and organic ligands to tune and enhance the magnetic response of molecular systems. The possibility of growing similar MOFs on other surfaces provides interesting prospect to tailor magnetic proximity effects and provide surfaces with ferromagnetic properties.^[^ ^119^, ^120^ ^]^ Importantly, the ferromagnetic states are indefinitely stable, so the magnetic MOF could act as PTM. However, challenges related to the system stability in air and to the low temperatures should be solved before introducing these systems in neuromorphic circuitry.

## 4 Integration of Responsive Molecules into Multiresponsive Memory Devices

**Figure 6:**
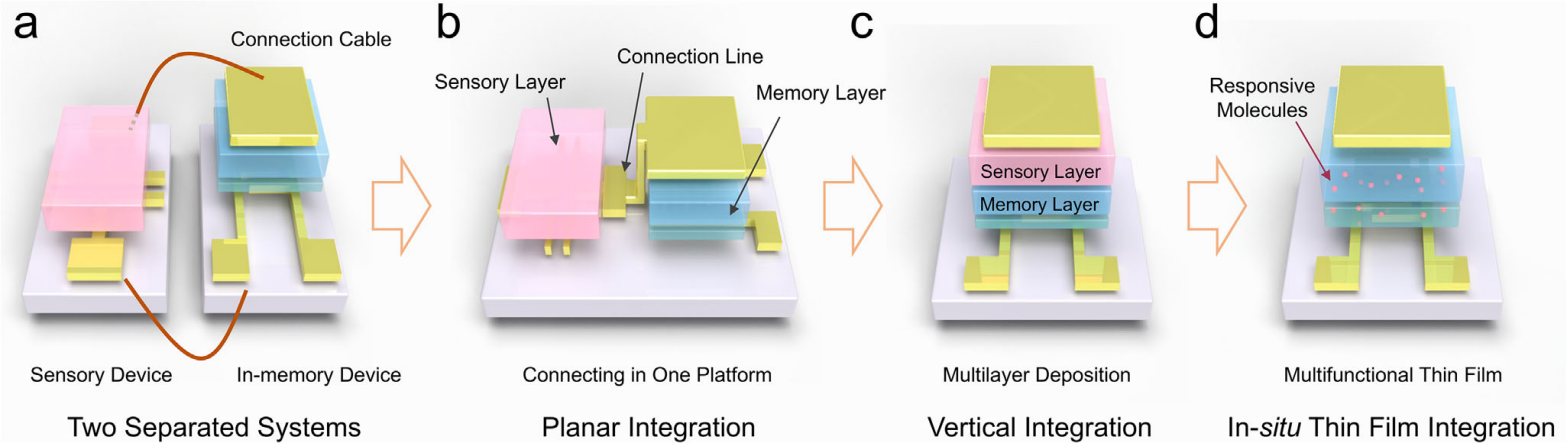
Development roadmap of in‐sensory computing system. a) Directly connecting the sensory device with in‐memory device, which needs the connecting cable between two different platforms. b) Planar integration of the sensory device and in‐memory device in same platform. c) Vertical integration of sensory material on the in‐memory device. d) Simple device architecture to achieve multifunction integration, which relied on the multifunctional thin films.

**Figure 7:**
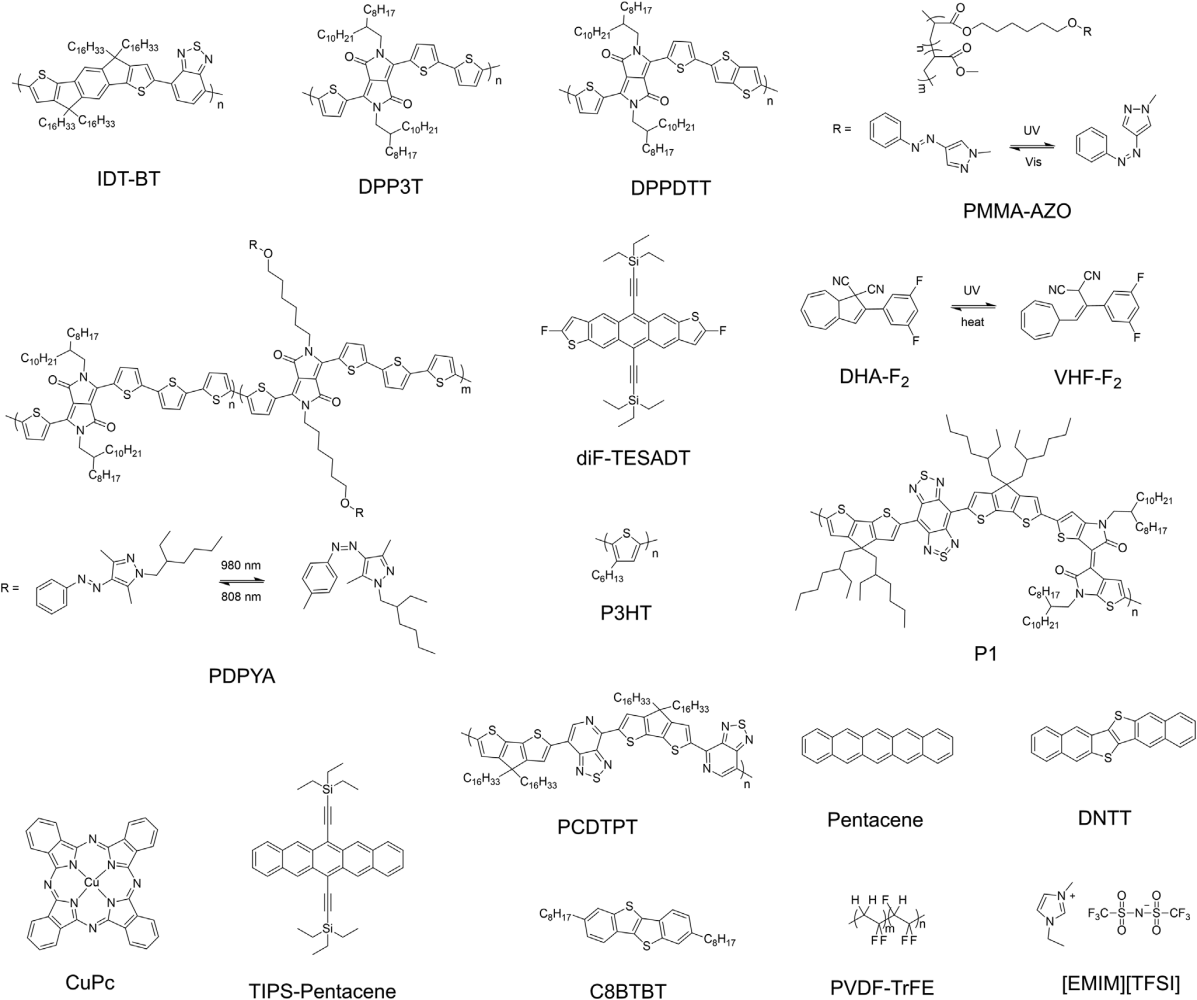
Chemical structure of molecules discussed in this Review.

The emerging “More than Moore” technologies have the ambition to solve the major bottlenecks associated with the miniaturization of transistor size, by mastering the integrated functional diversification into a single transistor.^[^ ^121^, ^122^, ^123^ ^]^ To improve the performance of chips and reduce the fabrication cost, in‐sensory computing technologies have been developed by exploiting four key approaches. i) In series connection of sensors with in‐memory devices. This strategy has been widely utilized in fundamental research studies due to its simplicity (**Figure** **6**a). However, the circuit resistance of connecting cables between two sensors increases the calculation speed and the energy consumption. ii) Fabrication on the same platform of two different devices connected by noble metal pad. This strategy enables to reduce the circuit resistance and accelerate data transmission (Figure 6b). However, despite these advantages, large‐scale integration remains challenging due to the complexity and compatibility of heterodeposition of different materials in two devices. iii) Vertical integration through the deposition of various functional layers in an ad hoc manner to empower the device to simultaneously perform sensory functions, storage functions, and computation functions (Figure 6c). For instance, the vertical interfacing of responsive materials between gate electrode and dielectric layer can be used to convert external stimuli into gate electrical spikes, thereby activating the memory dielectric layer. Yet, multilayer deposition technology still leads to high manufacturing cost and low production yield. iv) Multifunctional thin film assembly consisting in the incorporation of responsive molecules and active materials in the device. Among various architectures, it is the simplest and most low‐cost method to achieve in‐sensory computing. It operates via the modulation of the conductivity of channel layer or the dielectric constant of dielectric layer by exploiting a physical or chemical input to change the state of the embedded responsive molecules (Figure 6d). The chemical structure of the semiconducting molecules and polymers as well as responsive molecules discussed in this section is portrayed in **Figure** **7**.

### 4.1 Optical Memory Devices

Vision neural devices are crucial to accomplish AI tasks, especially for object recognition involving different colors, shapes, and movements. Compared with their inorganic counterparts, the organic responsive materials offer capability of tuneable bandgap and energy levels, enabling efficient light absorption across a broad range of wavelengths, spanning from ultraviolet (UV) to visible (vis) and near‐infrared (NIR) light. The following section introduces the reader to fabrication strategies for both nonvolatile and volatile optical memory devices based on the optically responsive molecules (**Table** **1**).

#### 4.1.1 Optical Nonvolatile Memory Devices

**Figure 8:**
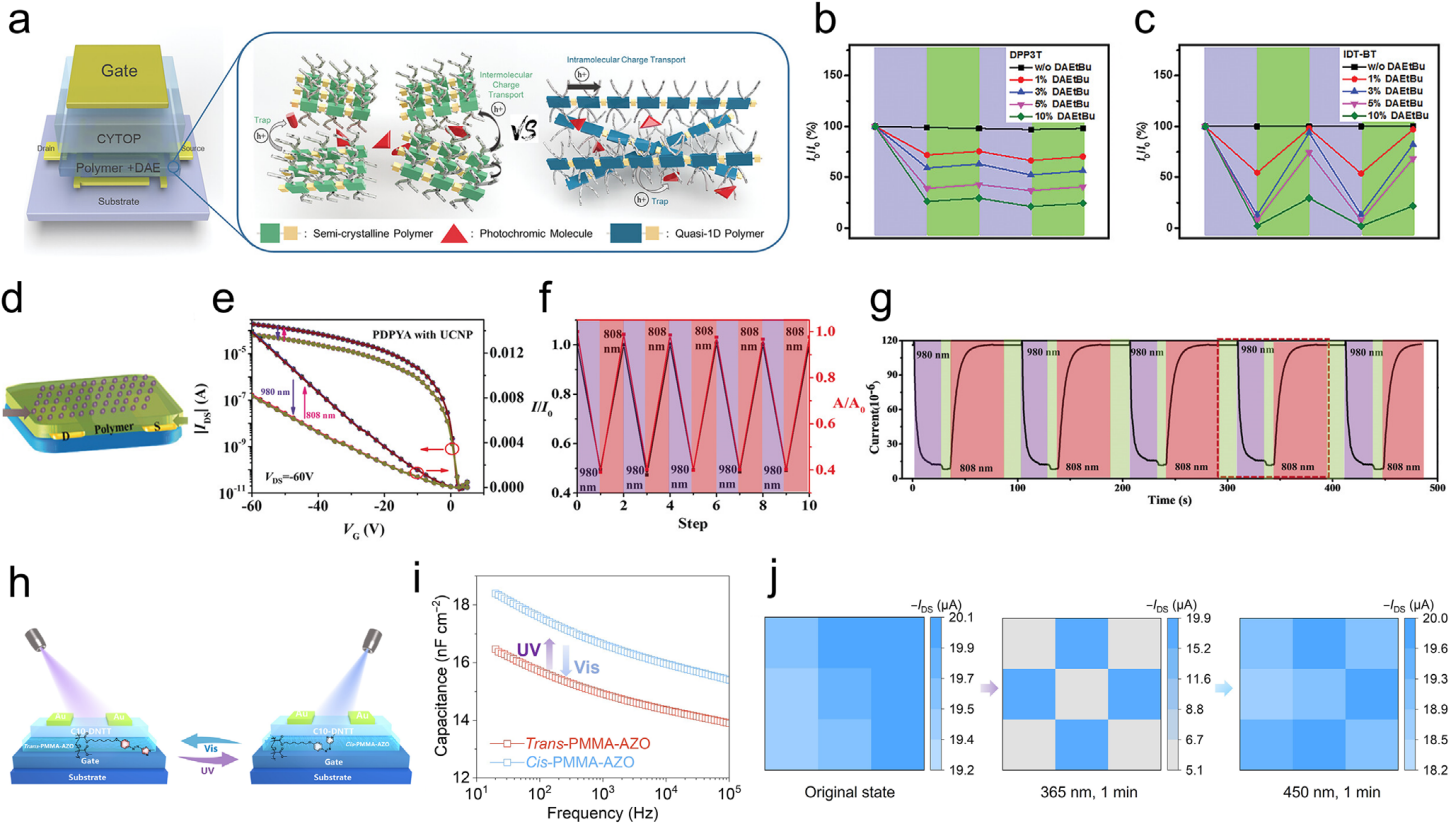
a) Schematic illustration of optically nonvolatile memory devices, including the molecular stacking comparison of semicrystalline versus quasi‐1D polymers. Reversible modulation of *I*_DS_ of devices with b) DPP3T/DAE blend film and c) IDT‐BT/DAE blend film upon cycling UV (312 nm, violet‐shaded areas) and visible light irradiation (530 nm, green‐shaded areas). (a–c) Reproduced with permission.^[^ ^126^ ^]^ Copyright 2023, Wiley‐VCH. d) Device structure of PDPYA:NaYF4:Yb,Tm‐based nonvolatile memory devices. e) Transfer curves of PDPYA:NaYF4:Yb,Tm‐based transistor after 5 cycles light irradiation of NIR light with wavelength of 980 and 808 nm, and f) the reversible current modulation upon alternating 980 and 808 nm light irradiation. g) Time‐dependence measurement of *I*_DS_ for PDPYA:NaYF4:Yb,Tm‐based transistor upon alternating 980 nm (20 s) and 808 nm (40 s) light irradiation. (d–g) Reproduced with permission.^[^ ^127^ ^]^ Copyright 2020, Wiley‐VCH. h) Schematic illustration of the PMMA‐AZO‐based optical memory transistor. i) *C*–*F* curves of the PMMA‐AZO film on the irradiation of visible and UV light, which correspond to *trans*‐state and *cis*‐state. j) The optical memory mapping based on 3 × 3 devices array with a UV light writing and visible light erasing process. (h–j) Reproduced with permission.^[^ ^128^ ^]^ Copyright 2024, AAAS.

The direct blending of photochromic molecules with organic semiconductors as the channel layer has been proven as a successful method to realize optical PTM.^[^ ^24^ ^]^ When irradiated at specific wavelengths, the DAE molecules can be toggled between the open‐ and the close‐ring states that possess different HOMO levels. This feature can be exploited for the light‐driven trapping and untrapping of the charge transport through an organic semiconductor matrix. This effect has been demonstrated extensively upon using semicrystalline polymer semiconductors whose electronic transport is characterized primarily by interchain processes.^[^ ^124^ ^]^ This issue implies the need of high degree of structural order to maximize the electrical performance of the devices. However, such required high structural order can have the countereffect to sterically hinder the photoisomerization of the embedded photochromic molecules.^[^ ^125^ ^]^ To overcome this problem, in a comparative study we demonstrated the key advantage of employing quasi‐1D polymers (i.e., IDT‐BT), characterized by charge transport dominated at the intramolecular level, instead of semicrystalline ones (i.e., DPP3T) (**Figure** **8**a). More importantly, charge transport in quasi‐1D polymers is much less sensitive to the degree of interchain order. Thus, the degree of crystallinity of the photoresponsive material embedded in the device has a limited influence on its output. In particular, when integrating blends of such two different polymer types with moderate amount of DAE as active layer in the transistors, a markedly different current output is observed, as revealed in Figure 8b,c.^[^ ^126^ ^]^ The device based on DPP3T can only be switched‐on but not switched‐off. Conversely, the device based on IDT‐BT exhibits both switch‐off and switch‐on behavior, demonstrating the superior photomemory capabilities of quasi‐1D polymer containing devices. The possibility to use only a small amount of DAE in the blend is important as it guarantees a minimal reduction of the device electronic function. Because of this reason, when introducing only 1% w/w of DAE molecules into the IDT‐BT thin film, high mobility exceeding 1 cm^2^ V^−1^ s^−1^, high photomodulation efficiency of 45.1% and high photorecovered efficiency of 98.1%, were simultaneously recorded. Such results are instrumental toward the development of multifunctional in‐sensory computing devices.

In addition to the blending approach of assembling optically switchable materials for optical PTM transistors (Figure 8d), Zhang's group showed that the photochromic functional moieties can also be grafted on the side chain of the semiconducting polymer.^[^ ^68^ ^]^ Toward this end, arylazopyrazole groups were grafted on the side chains of DPP (diketopyrrolopyrrole)‐based conjugated polymer, yielding PDPYA. The reversible *cis*–*trans* isomerization of the arylazopyrazoles can affect the backbone packing of the polymer chain, resulting in reversible tuning of intermolecular charge transporting properties, when irradiated with 348 and 470 nm light. Upon introducing the upconversion nanoparticles into the channel layer and utilizing the photothermal effect of conjugated polymers, the range of light responsive wavelengths of the device can be expanded to NIR light of 980 and 808 nm (Figure 8e,f). Real‐time measurement plotted in Figure 8g shows that the nonvolatile current recorded during 5 cycles light irradiation, displaying the excellent optically PTM.^[^ ^127^ ^]^

Recently, Hu's group reported a novel photochromic material with reversible dielectric constant (Figure 8h), which is obtained by tethering covalently arylazopyrazole moieties onto the backbone of polymethyl methacrylate (PMMA), yielding PMMA‐AZO. According to the capacitance–frequency (*C*–*F*) curves recorded from impedance measurement (Figure 8i), *trans*‐PMMA‐AZO and *cis*‐PMMA‐AZO thin film present capacitance of 16.5 and 18.4 nF cm^−2^ at a low frequency of 20 Hz, respectively. This change in capacitance originates from the remarkable dipole moment modification between the *trans* (3.7 D) to *cis* isomer (5.9 D), which can be triggered by exposure to UV light (365 nm) for 1.0 min. A nine‐PMMA‐AZO‐transistors‐based array was fabricated, whose drain–source currents were recorded and mapped in Figure 8j. Initially, the device currents ranged from 19.4 to 20.1 µA. However, after 1 min UV irradiation at 365 nm through an “X”‐image photomask, the currents of five organic FETs were synchronously reduced to ≈5.0 µA. Further exposure to 450 nm light can reconvert these memory currents back to the initial state.^[^ ^128^ ^]^

#### 4.1.2 Optical Volatile Memory Devices

**Figure 9:**
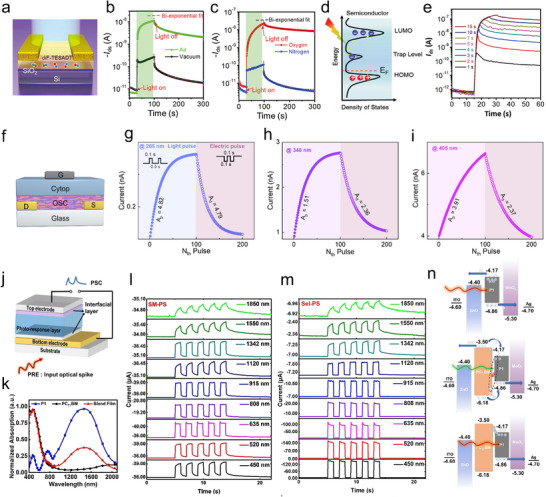
a) Device structure diagram of the diF‐TESADT‐based volatile memory device. The real‐time measurement PSC of devices upon the light irradiation, and under different atmospheres of b) vacuum/air and c) oxygen/nitrogen. d) Energy diagram of the diF‐TESADT and oxygen‐induced deep level, indicating the mechanism of volatile photoelectrons decay process. e) Dynamic PPC curve of device under light irradiation with different wavelengths. (a–e) Reproduced with permission.^[^ ^25^ ^]^ Copyright 2019, Wiley‐VCH. f) Device structure diagram of C8BTBT:PEA_2_PbBr_4_‐based optical memory device. LTP and LTD characterizations of devices responding to optically spiking with the wavelengths of g) 265, h) 340, and i) 405 nm and electric pulses. (f–i) Reproduced with permission.^[^ ^132^ ^]^ Copyright 2024, American Chemical Society. j) Device structure of the P1‐based two‐terminal synaptic device. k) Absorption spectra of P1 neat film, PCBM neat film, and P1:PCBM blend film. PSC of l) P1‐based and m) P1:PCBM‐based two‐terminal synaptic device under five times optical stimulus with different wavelengths. n) Volatile memory mechanism of P1‐based devices. (j–n) Reproduced with permission.^[^ ^133^ ^]^ Copyright 2023, Wiley‐VCH.

Persistent photoconductivity (PPC) is one volatile optical memory phenomenon, which is widely observed in metal oxide materials.^[^ ^129^, ^130^, ^131^ ^]^ Upon UV light irradiation, the conductivity of semiconductor is enhanced due to the increase in the concentration of free charges. Subsequently, the slow charge recombination provides a volatile nature to the recorded memory, thereby mimicking synaptic plasticity. Differently from metal oxide whose response is limited to UV light, volatile photoresponsive organic molecules and polymers exhibit light responsivity in a broader wavelength region. Jie's group proposed that oxygen molecules from air atmosphere act as deep defect levels in the energy bandgap of organic single crystal of 2,8‐difluoro‐5,11‐bis(triethylsilylethynyl) anthradithiophene (diF‐TESADT), providing metastable state for PPC property (**Figure** **9**a). The characterization of optical response was carried out under different atmospheres (Figure 9b,c), revealing that the PPC mechanism of these devices is due to oxygen‐induced deep trapping levels, which are storing the electron carriers. Upon irradiation with visible light at 575 nm, the diF‐TESADT crystals, being the channel materials in these devices, unveiled the occurrence of photo‐generated electron–hole pairs. Due to exposure of oxygen‐induced deep trapping levels, electrons were quickly transferred from polymer's LUMO level to the trapping levels and thereby stored, as illustrated in Figure 9d. In this case, the increased hole carriers’ concentration in channel layer is retained for minutes after the spiking light removal, leading to PPC behavior realization. The PPC decay property was determined by the light irradiation with different intervals, to mimic the synaptic behavior. The PPC curves in Figure 9e indicated that upon increasing the time of light irradiation the retention time of PSC can display sufficient STM to LTM transition.^[^ ^25^ ^]^

On the other hand, an artificial synapse responding both in the deep UV and visible light regions is crucial to realize neuromorphic facial recognition system. Hu's group demonstrated multidimensional deep ultraviolet optoelectronic synapses based on bulk heterojunction architecture, comprising a blend of organic semiconductor C8BTBT with the ultrawide bandgap perovskite materials PEA_2_PbBr_4_ (*E*_G_ = 3.03 eV) as the channel layer (Figure 9f). Linearity and conductance level of LTP/LTD are essential properties in neuromorphic computing because they affect the classification accuracy of neural networks. As plotted in Figure 9g–i, photonic pulses and electric pulses of 20 V were utilized for excitatory and inhibitory synaptic devices. Linearity of the potentiation and depression characteristics was extracted as 4.82/4.79, 1.51/2.36, and 3.91/2.37, when the light with wavelengths of 265, 340, and 405 nm were applied, respectively. Based on these synaptic parameters, a convolutional neural network architecture is tailored on this device by dividing the input data into three feature channels for compassion. Due to various response of device to the 265 nm, the grayscale conversion displays a significant difference between real person and mask person.^[^ ^132^ ^]^

A pioneering strategy to achieve optical volatile memory was proposed by Huang's group, by exploiting the unbalanced charge transport in light responsive molecules. An unprecedented artificial photonic synapse was demonstrated with responsivity to short‐wavelength infrared light. A donor–acceptor copolymer P1 with an ultranarrow bandgap of 0.69 eV was synthesized and constructed into a sandwich architecture (Figure 9j). As displayed in Figure 9k, the optical absorption spectra of P1 span across the entire visible region expanding to the short‐wavelength infrared light range. Herein, upon utilizing bare P1 as an active layer, the devices display full‐range PPC ranging from 450 to 1850 nm (shown in Figure 9l), exhibiting volatile memory behavior. The volatile memory of this device contributed to the unbalanced hole/electron mobility in P1, wherein the mobility ratio of holes and electrons was 3.45. Therefore, when the photonic input was absorbed by P1, the photo‐generated hole carriers drifted quickly to the anode, whereas, the particle photo‐generated electron carriers remained in the polymer matrix, leading to the volatile PPC property. More importantly, after blending n‐type materials PCBM with P1, selective wavelength responsive PPC behavior was recorded, as portrayed in Figure 9m. The ratio of hole/electron mobility of P1:PCBM blended film was determined as 1.04 through space charge limited current method. According to the schematic illustration in Figure 9n, when the visible light is irradiated on the device, P1 with PCBM acts as transport channel for photo‐generated holes and electron, respectively, leading to the balanced hole/electron charge transport. As a result, device based on P1:PCBM blended film exhibited only photodetection but not photomemory when exposed to visible light spiking input. Conversely, upon irradiating the device with short‐wavelength infrared light, only P1 absorbed the photons. Due to the LUMO of P1 being lower than that of PCBM, energy level barrier formed at the interface between P1 and PCBM. Herein, partial photogenerated electrons may be trapped in the P1 phase, thus resulting in the volatile photomemory current. By and large, the fine control over the balanced charge transport in blends of polymers with carefully engineered energy levels was demonstrated as a successful strategy toward selective phonic artificial synapse fabrication.^[^ ^133^ ^]^

Alongside PPC in conjugated molecules, also photochromic molecules characterized by metastable switching states are perfectly suitable to attain PPC behaviors. The Kraft's group reported a photochromic dihydroazulene (DHA)/vinylheptafulvene (VHF) molecular switch, demonstrating that the upon irradiation with UV light the DHA‐F_2_ derivative undergoes isomerization to form VHF‐F_2_ whereas reverse isomerization was obtained upon thermal stimuli. Nuclear magnetic resonance spectroscopy revealed that the complete thermal back‐switching from VHF‐F_2_ to DHA‐F_2_ occurs at room temperature within the timescale of 72 h. Although these molecules have been embedded into the memory transistor, the real‐time characterization of devices after UV spiked under room temperature is not reported.^[^ ^134^ ^]^ In principle, the well‐established SP/MC and azobenzene photoswitches also hold potential to achieve reconfigurable PPC behavior via the ad hoc design over their molecular structure (Table 1).

**Table 1:** Summary of optical memory devices.

Memory diversification	Active layer		Light wavelength	Refs.
	Conductivity materials	Optically memory materials		
Nonvolatile	DPPDTT	DAE‐tBu	530 and 312 nm	[125]
	IDT‐BT	DAE‐tBu	530 and 312 nm	[126]
	PDAZO	PDAZO	365 and 470 nm	[68]
	PDPYA	PDPYA:NaYF4:Yb,Tm	980 and 808 nm	[127]
	C10‐DNTT	PMMA‐AZO	365 and 450 nm	[128]
	ICBA	DAE‐FN	540 and 320 nm	[135]
	azo‐tz‐PEDOT:PSS	azo‐tz‐PEDOT:PSS	365 nm	[67]
Volatile	diF‐TESADT	diF‐TESADT with oxygen trap	575 nm	[25]
	C8BTBT	PEA_2_PbBr_4_ and C8BTBT	265, 340, and 405 nm	[132]
	P1	P1	From 450 to 1850 nm	[133]
	P1:PCBM	P1:PCBM	From 1342 to 1850 nm	[133]
	TPP	DNTT	450 nm	[136]
	Pentacene	IR780	780 nm	[137]
	C8BTBT	PSBOTz	450, 525, and 620 nm	[138]
	PDPP4T	Chlorophyll/PDPP4T blend film	430 nm	[139]
	PDPPBTT	PDPPBTT/SnO_2_ bilayer	808 nm	[140]

### 4.2 Mechanical Memory Devices

Materials capable of responding to mechanical stimuli can be exploited for both tactile and auditory devices, which detect the touch of skin and ear the sound waves, respectively. Unlike inorganic piezoelectric materials, elastomeric polymers are well‐suited as mechanical responsive molecules for the fabrication of pressure sensor and artificial skin, in view of their excellent flexibility and high durability. The stress stimulus in elastomeric polymers can be converted into electrical signal by taking advantage of the piezoresistive, capacitive, and triboelectric nature of the materials. By combining electrical memory storage molecules with elastomeric molecules, it is possible to realize mechanical memories upon using the fabrication strategies introduced in following section (**Table** **2**).

#### 4.2.1 Mechanical Nonvolatile Memory Devices

**Figure 10:**
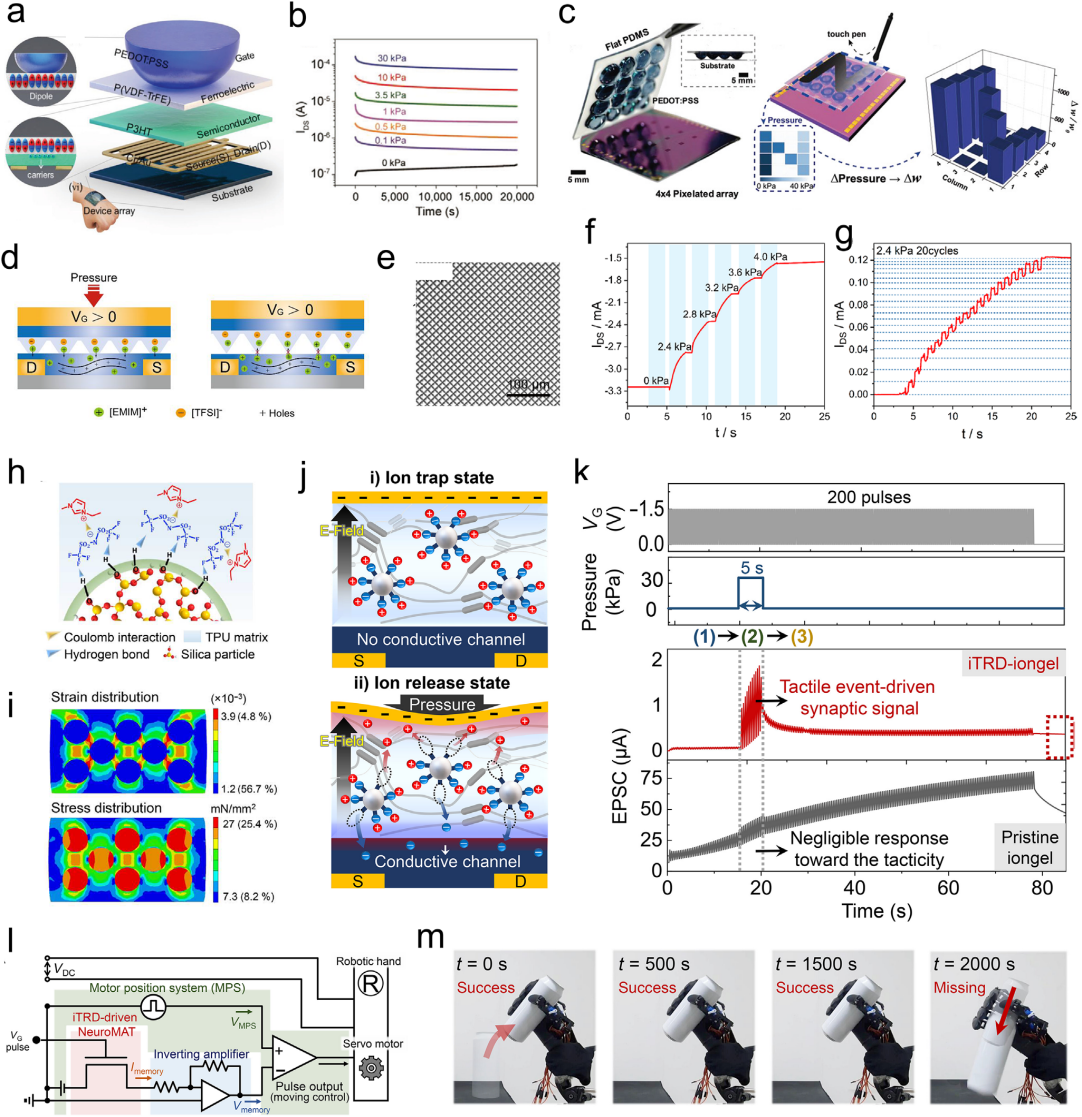
a) Schematic illustration of the mechanical nonvolatile memory device. b) Dynamics retention characterizations of device after the pressure spiking ranging from 0.1 to 30 kPa. c) Photograph of 4 × 4 pixelated mechanical memory devices array written by a commercial touch pen with an alphabet “N” pattern. (a–c) Reproduced with permission.^[^ ^141^ ^]^ Copyright 2020, Wiley‐VCH. d) Schematic diagram of PTM mechanism of devices. e) SEM images of microstructure surface of nanopattern ion‐gel. f) Real‐time *I*_DS_ curves of device after different pressure spikes. g) Multilevel nonvolatile record of device upon 2.4 kPa pressure is applied. (d–g) Reproduced with permission.^[^ ^142^ ^]^ Copyright 2024, American Chemical Society. h) Schematics of hydrogen bond between silica particles and [EMIM]^+^. i) Simulation results of effective stress region of ion‐gel under applied pressure. j) Illustration of work principle of nonvolatile memory device. k) Real‐time characterization of devices under dynamic tactile stimulation of 10, 30, and 50 kPa. l) Circuit diagram of artificial neural system which included nonvolatile memory device, inverting amplifier, motor position system, and anthropomorphic robotic hand. m) Photographic images neural system showing the nonvolatile gripping motion after pressure spiked. (h–m) Reproduced under the terms of the CC‐BY license.^[^ ^28^ ^]^ Copyright 2023, AAAS.

Piezoresistive sensors can be incorporated into transistors by exploiting mechanical responsive conducting materials as the gate‐terminal. Subsequently, upon introducing a memory dielectric layer one can integrate mechanical sensory and in‐memory function, which is caused by the memory field‐effect modulation on channel layer after pressure spiking. For example, Park's group reported an artificial tactile synapse, based on a dome‐shaped top‐gate electrode of PEODT:PSS‐covered‐polydimethylsiloxane (PDMS) and ferroelectric dielectric molecules of P(VDF‐TrFE). As shown in **Figure** **10**a, the contact area of upper gate electrode with the bottom ferroelectric layer can be controlled by the magnitude of the external stimuli. Herein, in presence of a pressure spike input, the gate electrical field is successfully applied on the P(VDF‐TrFE) due to the deformation of PDMS, thereby resulting in the molecular dipole alignment of P(VDF‐TrFE). Upon removal of the pressure stimulus, the molecular dipole alignment is retained thus providing a continuous gate effect on the channel layer, which is reflected in the device's drain–source current (*I*_DS_) output. The hysteresis transfer curve in Figure 10b reveals that there are two distinct ON and OFF states with *I*_ON_/*I*_OFF_ ratio of ≈10^3^ at *V*_G_ = 0 V, originating from the fully saturated up and down remnant polarization domains in the P(VDF‐TrFE) layer, respectively. Various pressure levels ranging from 5 × 10^−2^ to 30 kPa were applied on the device, leading the multilevel current storage with excellent PTM of ≈20 000 s. In particular, as shown in Figure 10c and 4 × 4 pixelated array of tactile memory devices was fabricated for the letter recognition. An “N” pattern spiking written by touch pen was applied on tactile device array, leading to synaptic weight changing of devices. The different weight encoding of each device can be used to identify the users based on their different writing styles.^[^ ^141^ ^]^

Upon using nanopatterning technology, one can exploit ion‐gel with the function of both dielectric layer and pressure sensing as the capacitive mechanical sensor. For example, the Yan's group reported an organic electrochemical transistor, whose device structure is displayed in Figure 10d. The morphology of ion‐gel used in this work was assessed by scanning electron microscope (SEM) as shown in Figure 10e. The pyramid shape of ion‐gel enables the modulation of distance between the gate electrode and channel layer under variable pressure stimulus. Initially, in absence of applied pressure the capacitance of the pyramid‐patterned ion‐gel is low because the distance between the two capacitor plates is large. Upon increasing pressure, the capacitor plates get closer and the moving ions immersed into PEDOT:PSS enables conductivity modulation. Once the applied pressure is removed, the connection of ion‐gel and PEDOT:PSS is withdrawn, with the ions remaining in channel layer for PTM. Dynamic characterization of the device was carried out and depicted in Figure 10f,g. Stable current storage was recorded upon applying a pressure from 2.4 to 4 kPa. Importantly, highly linear multilevel memory facilitation was achieved upon repeated pressure stimulus of 2.4 kPa for 20 times.^[^ ^142^ ^]^

One innovative strategy to develop multifunctional thin films was demonstrated by Kim and co‐workers, by developing an ion‐gel capable of simultaneously sensing and storing data. The mechanical PTM mechanism of the investigated ion‐gel of thermoplastic polyurethane (TPU) polymer matrix is depended on the ion trap and release dynamics, which is achieved by the strong dipole–dipole interaction between the hydrogen (H) atom exposed on the silica microparticles and fluorine (F) side‐groups of the polyurethane, ruling the movement of ions of [TFSI]^−^, as portrayed in Figure 10h. The silica microparticle and TPU matrix is endowed with various elastic moduli of 30 GPa and 10 MPa, respectively. In Figure 10i, the finite element calculations results indicate that the mechanical stress applied on the ion‐gel was mainly localized at the silica microparticle and TPU matrix interface, herein increasing the distance between [TFSI]^−^ and the silica surface, thereby effectively reducing the binding energy of the H···F bond. Time‐dependent measurements of devices with and without silica microparticle are plotted in Figure 10k. They reveal that in absence of silica microparticle for ions traps, the moving ions migrate into the channel layer of DPPDTT under the effect of the gate electric field. The pressure spikes input determined a negligible current modulation, indicating the unobvious change of ions distribution in bare ion‐gel. Conversely, the integration of silica microparticle into ion‐gel makes it possible to optimize ions distribution. Under the effect of an electric gate, since most of the [TFSI]^−^ are bonded, the *I*_DS_ remains at low magnitude (Figure 10j). When pressure spike is applied on the device, trapped ions are released, getting into the channel layer of DPPDTT, ultimately leading to enhancement of *I*_DS_ with PTM of 3680 s due to electrochemical reaction. Furthermore, a higher *I*_DS_ was achieved upon applying a higher pressure, a scenario that can be attributed to the increase in the stress distribution and magnitude near the silica microparticles as the applied pressure increased. As proof‐of‐concept, this mechanical nonvolatile memory device displays a successful application for memory‐based human motion, which is achieved by integration with robotic hand system including inverting amplifier, moving controller and servo motor (Figure 10l,m). When robust pressure is applied on the device as training process, the robotic hand system could consistently and reliably perform gripping and lifting motions over 2000 s. This result implies that the development of mechanical nonvolatile memory devices can enable reliable emulation of human motion system with ability of training‐to‐learning.^[^ ^28^ ^]^

#### 4.2.2 Mechanical Volatile Memory Devices

**Figure 11:**
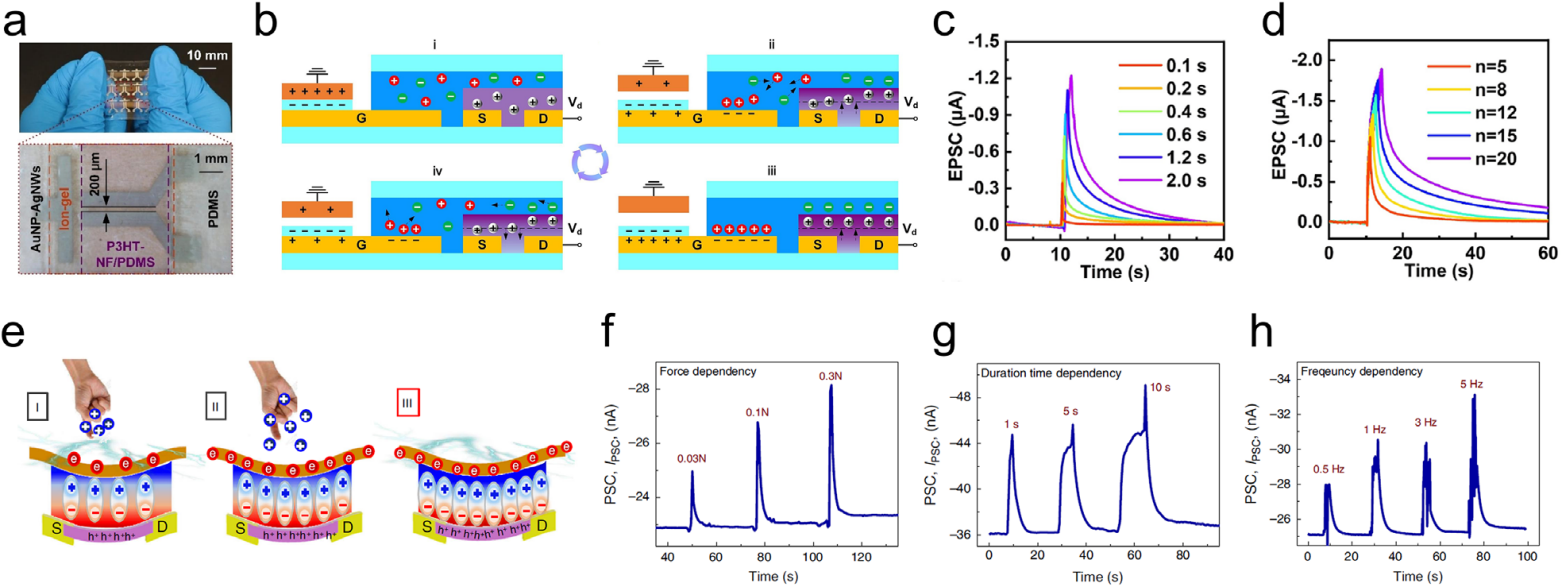
a) Photograph of stretchable mechanical memory devices. b) Structure diagram and work principle of mechanical volatile memory device. Real‐time measurement of c) spike‐timing‐dependent plasticity and d) spike‐timing‐dependent plasticity. (a–d) Reproduced with permission.^[^ ^143^ ^]^ Copyright 2024, Elsevier. e) Scheme diagram of synaptic tactile sensory organ, wherein dipole switching in the ferroelectric layer after tactile stimulation. f) Spike‐strength‐dependent synaptic plasticity, g) spike‐duration‐dependent synaptic plasticity, and h) spike‐rate‐dependent synaptic plasticity characterizations of mechanical volatile memory devices. (e–h) Reproduced under the terms of the CC‐BY license.^[^ ^26^ ^]^ Copyright 2020, Springer Nature.

Triboelectric nanogenerator (TENG) is a self‐power device which can generate electrical energy from mechanical motion or pressure via the triboelectric effect. It can be found in triboelectric molecules which are capable of responding to a mechanical stimulus. Zhang and co‐workers integrated a stretchable organic chemical transistor with a TENG based on the triboelectric responsive molecules of PDMS (**Figure** **11**a). The device structure and working principle are portrayed in Figure 11b. The PDMS is covered directly by the ion‐gel and copper deposited as external triboelectric film. Triboelectrification occurs at the interface between the copper and PDMS film, leading to the positive and negative charges accumulated on copper film and PDMS film, respectively. When the copper film moves away, negative charges on PDMS film lose the screened effect, thereby inducing the opposite charges on gate electrode. Subsequently, the gate electrode powers anions immersed into channel layer of P3HT, operating as mechanical induced current. Upon reapproaching the copper film, the immersed anions withdraw slowly to the ion‐gel thereby providing a volatile memory *I*_DS_. A single mechanical stimulation with displacement distance (*D*) of 2 mm and various duration time were applied on the devices. As plotted in Figure 11c, the PSC increased from −0.3 to −1.2 µA, revealing the spike‐timing‐dependent plasticity. Similarly, a series of mechanical stimulation with times were applied on the devices, as shown in Figure 11d, which presented spike‐times‐dependent plasticity. More importantly, the prolonged retention time was observed in the higher PSC, mimicking the synaptic function of STM to LTM transition.^[^ ^143^ ^]^

The pressure applied by fingers can be exploited to empower the triboelectric‐capacitive coupling effect, inducing the alignment of dipoles in the ferroelectric gate dielectric. Lee et al. report a flexible, artificial, intrinsic synaptic tactile sensory organ by using Ni, barium titanate nanoparticles/PVDF‐TrFE, and pentacene as gate electrode, triboresponsive dielectric layer and semiconductor layer, respectively (Figure 11e). The touching spike applied onto gate electrode enables the alignment of dipoles in the ferroelectric materials and provides the gate electrical field for modulating the channel conductivity. Once the spike is removed, the alignment of dipoles is retained thereby providing the memory current. The artificial synaptic behavior of these devices was investigated upon various touch stimuli with different strengths, durations, and frequencies. As shown in Figure 11f–h, these results indicated the successful transition from STM to LTM (Table 2).^[^ ^26^ ^]^

**Table 2:** Summary of mechanical memory devices.

Memory diversification	Active layer		Responding pressure	Refs.
	Conductivity materials	Mechanical memory materials		
Nonvolatile	P3HT	Dome‐shaped PEDOT:PSS/P(VDF‐TrFE) bilayer	0.1 to 63 kPa	[141]
	PEDOT:PSS	Pyramid‐patterned ion‐gel of (P(VDF‐HFP):[EMIM][TFSI]	2.4 to 4.0 kPa	[142]
	DPPDTT	[EMIM][TFSI]:silica microparticles:polyurethane	4 to 50 kPa	[28]
	WSe_2_	FEP‐based TENG with floating gate of graphene	*D* from 10 to 30 µm	[144]
Volatile	P3HT	PDMS‐based TENG with ion‐gel of PVDF‐HFP:[EMIM][TFSI]	*D* of 2 mm	[143]
	Pentacene	BaTiO_3_ nanoparticles:P(VDF‐TrFE)	≈1 kPa	[26]
	P3HT	PTFE‐based TENG with ion‐gel of PVDF‐HFP:[EMIM][TFSI]	D of 2 mm	[145]
	P3HT	Pyramid‐patterned ion‐gel of (P(VDF‐HFP):[EMIM][TFSI]	0.5 to 7.5 kPa	[146]

### 4.3 Electrochemical Memory Devices

The biochemical stimulus is invisible and intangible yet in some cases it can become extremely dangerous. Odor gas molecules in the atmosphere and acid molecules in rotten food can be harmful thus their detection is essential. To emulate the function of olfactory neurons and gustatory neurons, electrochemical transistors represent as an ideal platform, wherein the conductivity of organic semiconductors can be modulated by polar biochemical signals, relying on redox reactions.^[^ ^147^, ^148^, ^149^, ^150^ ^]^ To achieve in‐sensor computing, the strategies of storing the sensory biochemical signal with nonvolatile and volatile memory are introduced in the following section (**Table** **3**).

#### 4.3.1 Nonvolatile Electrochemical‐Responsive Devices

**Figure 12:**
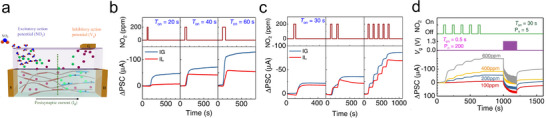
Schematic illustration of a) artificial olfactory synapse. Compared characterization of b) spike‐duration‐dependent synaptic plasticity and c) spike‐times‐dependent synaptic plasticity, where the IL and IG represented ion‐liquid and ion‐gel. d) Responsive current under the excitatory stimuli of gas molecules with different concentrations and inhibitory stimuli of gate voltage. (a–d) Reproduced under the terms of the CC‐BY license.^[^ ^29^ ^]^ Copyright 2023, Springer Nature.

Organic electrochemical transistors are electronic devices holding a key advantage: both ion‐liquid and ion‐gel can operate as electrolyte allowing ions and molecular migration. However, the distribution of gas molecules inside these two electrolytes is different, resulting in various memory retention times. Lee and co‐workers compared the performances of ion‐liquid and ion‐gel in NO_2_ driven responsive memory in an organic chemical transistor (**Figure** **12**a). The ion‐gel was prepared by blending an ionic liquid of [EMIM][TFSI] with PEGDA monomer, and a photoinitiator with ratio of 90:8:2, and subsequently cross‐linked under the UV light irradiation. Upon device exposure to NO_2_ atmosphere, the NO_2_ molecules diffused into electrolyte and reacted with the [EMIM]^+^ cation due to their unpaired electron. Herein, the remainder anion of [TFSI]^−^ moves into channel layer, leading to the gate effect for the PEDOT:PSS and enhancing the PSC. Time‐dependent measurements of the current response to NO_2_ gas exposure for both ion‐liquid and ion‐gel based devices were recorded, by changing the durations and times of exposure, as portrayed in Figure 12b,c. It was found that ion‐gel‐based devices exhibit a higher synaptic weight change and a prolonged retention time, compared to ion‐liquid‐based devices. The NO_2_ molecules induced PTM due to the slower redistribution of ions inside the polymer matrix when compared to the ion‐liquid. On the same time, multilevels NO_2_‐spiked PTM were recorded when NO_2_ gas exposure was repeated, allowing the application for the in‐sensor computing. Desorption of gas molecules in the ion‐gel electrolyte is also important for the inhibition of PSC. The positive gate voltage of 1 V was utilized to achieve long‐term depression (Figure 12d). The higher level of excitatory PSC caused by the higher concentration gas stimulus (600 ppm) remains the higher level of inhibitory PSC compared with that spiked by lower concentration gas (100, 200, and 400 ppm), which proves the anti‐forgetting ability of PTM.^[^ ^29^ ^]^

#### 4.3.2 Volatile Electrochemical‐Responsive Devices

**Figure 13:**
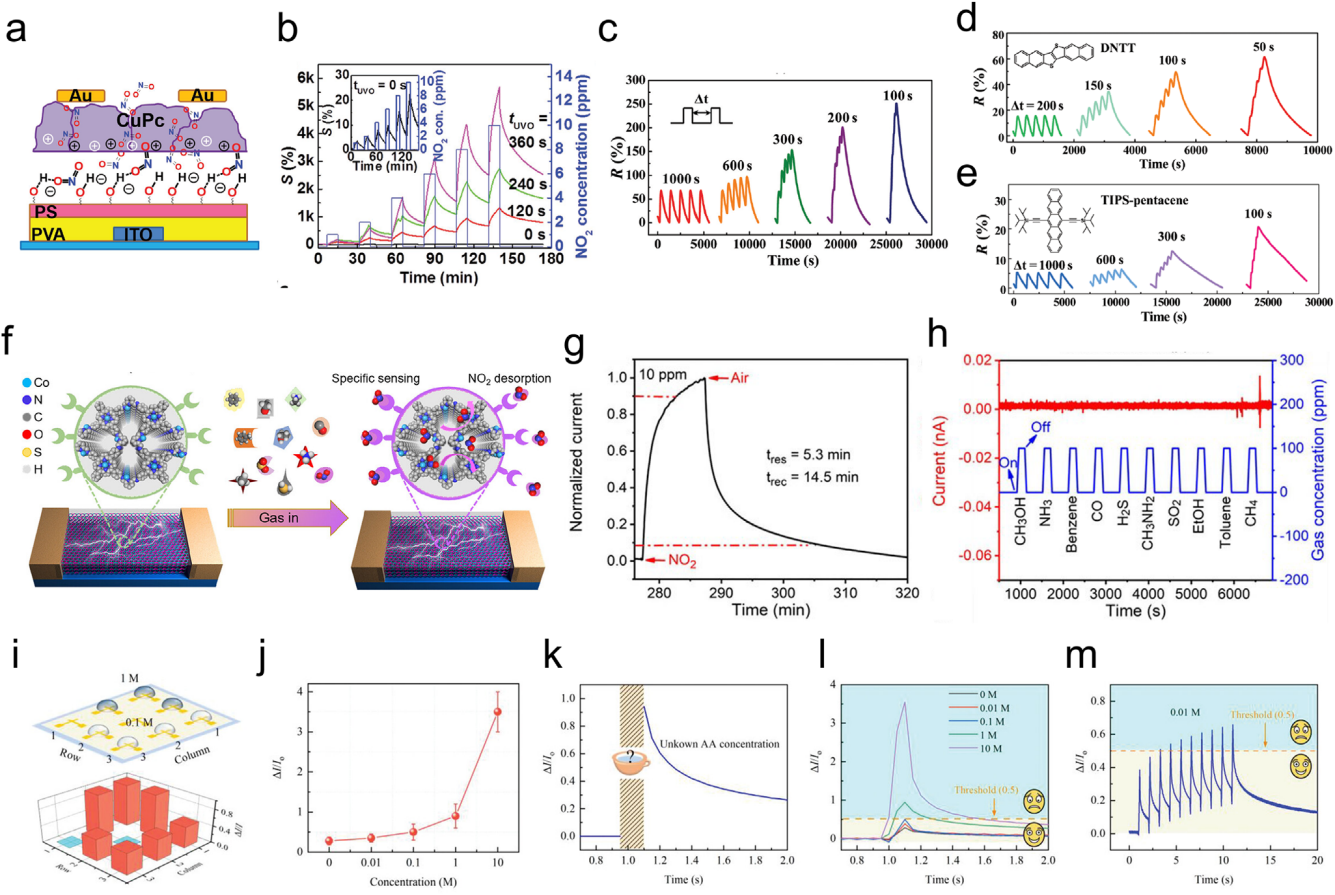
a) Schematic representations of the NO_2_ diffusion process through the channel layer and adsorption process with the UVO‐treated PS surface. b) Dynamic sensitivity of gas volatile memory devices with different UVO‐treated times. (a,b) Reproduced with permission.^[^ ^151^ ^]^ Copyright 2017, Wiley‐VCH. Gas responsivity of gas volatile memory devices under gas spikes with different time interval when c) PCDTPT, d) DNTT, and e) TIPS‐pentacene utilized as channel layer, respectively. (c–e) Reproduced with permission.^[^ ^152^ ^]^ Copyright 2019, Royal Society of Chemistry. f) Device structure diagram of gas volatile memory devices based on COF materials. g) Time‐dependence measurement and h) selective sensitivity of COF‐based gas volatile memory. (f–h) Reproduced with permission.^[^ ^153^ ^]^ Copyright 2022, Wiley‐VCH. i) Scheme of the arrayed artificial tongues responding to 0.1 and 1 m of acetic acid for taste mapping. j) Average of responsive current (Δ*I*/*I*_0_) upon various acetic acid concentrations, which are calculated from at least five devices. k) The current change in response to voltage stimulation with an unknown acetic acid concentration. l) Real‐time characterization for artificial analysing the unknown acetic acid concentrations. m) Device's current response to a continuous stimulus at a constant acetic acid concentration of 0.01 m, which can reach threshold value after the training process. (i–m) Reproduced with permission.^[^ ^27^ ^]^ Copyright 2022, Wiley‐VCH.

Organic semiconductors can be easily doped via the polar gas molecules, leading to changes in their conductivity, thereby acting as gas‐responsive materials. For such application, the FET geometry plays a key role. The use of ion‐gel gate covered by the organic semiconductor implies a slow diffusion of the gas molecules through the polymer layer, limiting both the responsive and recovery time. Conversely, in bottom‐contact top‐gate architecture, the behavior of volatile gas responsive memory is faster. Facchetti and co‐workers have reported NO_2_ gas olfactory devices based on a 10 nm thick film of copper phthalocyanine (CuPc), which is deposited on ITO/PVA/PS substrate (**Figure** **13**a). In this work, the surface of polymeric gate dielectric of polystyrene was modulated by UV–ozone (UVO) treatment to increase surface trap density, which was assessed by both the X‐ray photoelectron spectroscopy and Fourier transform infrared spectroscopy measurement. Herein, the oxygenated polar functionalities can efficiently adsorb polar gas molecules via hydrogen bonding or van der Waals interactions, thus inducing positive charging of the semiconductors by polarization effects. Real‐time NO_2_ exposure dynamic sensitivities of devices before and after UVO treatment were plotted in Figure 13b for comparison. Increasing the UVO treatment time from 0 to 360 s, the sensitivity of devices enhanced significantly from 4% to 3200%, along with displaying a volatile memory process.^[^ ^151^ ^]^ To evaluate the universal nature of organic semiconductors for the NO_2_ molecules inducing memory effects, Liu and co‐workers fabricated thin film transistor based on the PCDTPT polymer and small molecules of DNTT and TIPS‐pentacene. As shown in Figure 13c–e, all these devices exhibit volatile memory with synaptic property of STM to LTM transition.^[^ ^152^ ^]^

Another type of storage process involves the adsorption of gas molecules. The optimal molecular materials for this process are covalent organic frameworks (COFs), in view of their large surface area, highest porosity, structural robustness, and programmable chemical structure. Such characteristics make therefore COFs ideal active components for in‐sensor devices. Lan and co‐workers reported ultrathin nanosheets 2D‐COF materials based on porphyrin–Co building block, which display strong π–π interactions.^[^ ^153^ ^]^ Such COF's ultrathin films were integrated in two‐terminal devices, which displayed a strong response to NO_2_ (Figure 13f). Figure 13g shows that upon exposure to 10 ppm NO_2_ the device's current increases gradually and with a persistent memory time of 5.3 min. Notably, the high selectivity for NO_2_ was demonstrated as the sensor showed negligible response to other molecular gas (Figure 13h). Such a proof‐of‐concept provides evidence that view of their cage‐like morphology with programmable chemical structure, COF materials hold huge potential for application as (bio)chemical in‐sensory computing device. However, the low conductivity and batch‐to‐batch variation of COF materials still limit their implementation in electronic devices.^[^ ^154^ ^]^ Recent findings on the synthesis of single‐crystalline sp^2^ carbon‐linked COFs via imine‐to‐olefin transformation open a new avenue for the development of COFs with radically enhanced electronic functions.^[^ ^155^ ^]^

In addition to artificial olfactory devices, artificial gustatory devices can also be realized by using the organic electrochemical transistor platform. Inspired by human taste perception, Liu et al. demonstrated gustatory memory device to detect acetic acid in an electrolyte‐gated transistor with vertical channel. As depicted in Figure 13i, a 3 × 3 device array can be operated for taste mapping, wherein the cross‐linked P3HT polymer was vertically stacked on the source/drain electrodes and the ionic liquid containing two different concentrations (0.1 and 1 m) was wrapped on channel layer. The current changes in response to acetic acid at different concentrations are displayed in Figure 13j with the sensitive range spanning from 0.01 to 10 m. Interestingly, these devices can be employed for acidity discrimination by a setting threshold of current change ratio (Δ*I*/*I*_0_) at 0.5. In particular, when a stimulus with acid content higher than 0.1 m is applied, the change in PSC ratio exceeds 0.5, which can be interpreted as “painful expression” (Figure 13k,l). Caused by synaptic function of STM to LTM transition, rehearsal of stimulus with a low acid content of 0.01 m can also provide a “painful expression” (Figure 13m). These findings highlight a promising approach for advancing human–robot interactions, paving the way for more intuitive and responsive systems that can closely mimic human behavior and adaptability (Table 3).^[^ ^27^ ^]^

**Table 3:** Summary of electrochemical memory devices.

Memory diversification	Active layer		Responding targets	Refs.
	Conductivity materials	Electrochemical memory materials		
Nonvolatile	PEDOT:PSS	Ion‐gel of PEGDA:[EMIM][TFSI]	NO_2_	[29]
Volatile	CuPc	UVO‐treated PS	NO_2_	[151]
	PCDTPT	PCDTPT	NO_2_	[152]
	Co‐TPCOF	Co‐TPCOF	NO_2_	[153]
	P3HT	Ion‐liquid of [EMIM][TFSI]	Acetic acid	[27]
	CuPc:pentacence	CuPc:pentacene bilayer	NO_2_	[156]
	PTDPPSe‐5Si	PTDPPSe‐5Si	NH_3_ and NO_2_	[157]
	TIPS‐pentacene	TIPS‐pentacene	NO_2_	[158]
	Pentacene/IGZO	Pentacene	NH_3_	[159]
	PEDOT:PSS	Porous electrolyte of PVDF‐*co*‐HFP:[BMIM][TFSI]	H_2_S	[160]
	SnO_2_ NWs	Ion‐gel of chitosan	Na^+^	[161]
	COF‐TXDBA	COF‐TXDBA	H_2_O	[162]
	COF‐DC‐8	COF‐DC‐8	NH_3_, H_2_S, NO, and NO_2_	[163]

## 5 Nonvolatile Memory and Volatile Memory Integration

In the human brain, learning and training process rely on the dynamic interplay between STM and LTM. Conversely, PTM is crucial for storing information over extended periods, it suffers from inference process and decision making after suitable training. Herein, the joint effect of volatile and nonvolatile memory enables AI systems to tackle complex tasks that require both immediate responses and long‐term strategic planning, closely mirroring the brain's natural processes. For example, Luo and Yu have proposed the nonvolatile and volatile memory device integration within each junction point in a crossbar array architecture for deep neural networks. During the training step, the modulation of synaptic gradients was implemented on volatile memory device. Conversely, after certain training batches, synaptic weights access the threshold to be transferred into nonvolatile memory device, thus exploiting the inference process. Such nonvolatile and volatile memory device integration architecture allows the storage of key information upon simultaneously saving energy consumption.^[^ ^164^, ^165^ ^]^

**Figure 14:**
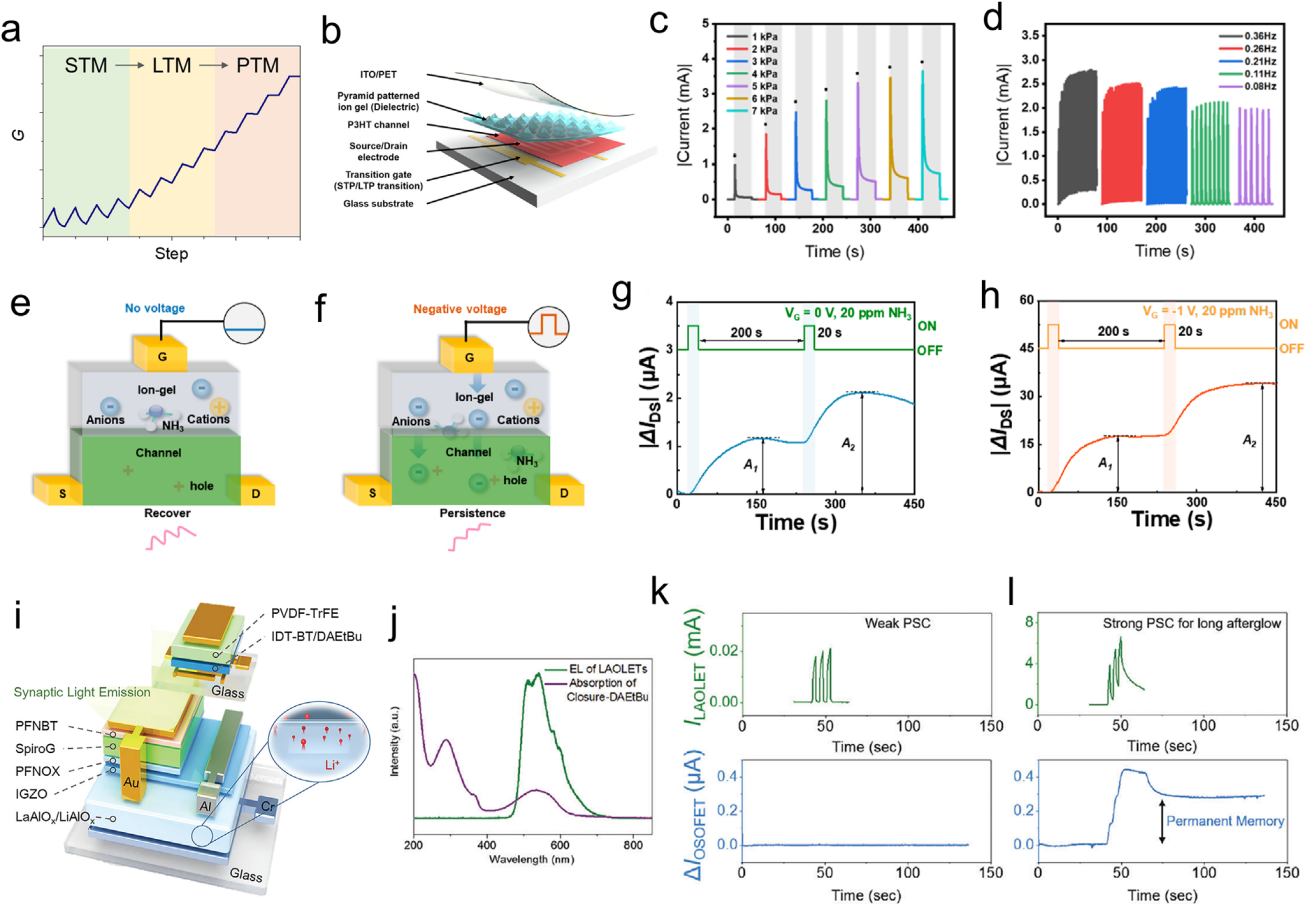
a) Schematic illustration of the in situ STM‐to‐LTM‐to‐PTM step‐by‐step transition. b) Schematic illustration of bi‐modal artificial tactile device. c) Real‐time characterization of nonvolatile memory when *V*_G_ of 0 V and pressure with different strengths. d) Real‐time characterization of volatile memory when *V*_G_ of 1 V and pressure with different frequency were applied. (b–d) Reproduced with permission.^[^ ^146^ ^]^ Copyright 2020, American Chemical Society. e,f) Device structure scheme for working principal interpretation of bi‐modal artificial olfactory device, when *V*_G_ = 0 V and *V*_G_ = −1 V was applied, respectively. Time‐dependent measurement for the g) volatile memory and h) nonvolatile memory. (e–h) Reproduced with permission.^[^ ^168^ ^]^ Copyright 2024, American Chemical Society. i) Schematic illustration of memory optocoupler, where the bottom panel is a synaptic light‐emitting transistor and the upper panel is an optical memory transistor. j) Normalized spectra of electroluminescence of display device and absorption spectra of responsive molecules in sensory devices. Time‐dependence measurement of memory optocoupler, when three times of k) weak stimulus of (*V*_G2_ = 2 V) and l) strong stimulus (*V*_G2_ = 12 V) applied. (i–l) Reproduced with permission.^[^ ^169^ ^]^ Copyright 2024, Wiley‐VCH.

The coexistence of nonvolatile memory and volatile memory has been observed in some inorganic memristors, which mainly depend on the formation of conductive filaments when the spiking voltage exceeds of threshold. A simple sandwich memristor of Pt/HfO_2_/Ti was utilized to realize the in situ volatile memory to nonvolatile memory transition. A series of voltage pulses with an amplitude of −7 V were applied as the presynaptic spiking. As revealed in the conductance plotted in **Figure** **14**a, the device at the low resistance state presents the volatile memory during the first few pulses. Conversely, after several spiked rehearsal, a nonvolatile memory behavior is observed with ultralow resistance states and a retention time over 10^5^ s, evidencing the LTM to PTM transition function.^[^ ^166^, ^167^ ^]^

Hitherto, realizing in situ LTM to PTM transition in organic memory devices is still a great challenge. However, based on organic electrochemical transistors, bimodal application of volatile memory and nonvolatile memory can be achieved with the assistance of gate voltage. Park and co‐workers reported an artificial tactile device based on pyramid‐pattern ion‐gel and P‐type channel layer (Figure 14b). As shown in Figure 14c, upon applying pressure spiked with different strengths to the device in the absence of gate voltage, various levels of PSC current can be recorded as PTM. However, upon applying a gate voltage of 1 V and pressure stimulus, PSC exhibits typical paired‐pulse facilitation behavior, which is influenced by the frequency of stimulus (Figure 14d).^[^ ^146^ ^]^ Similarly, Huang and co‐workers proved the bimodal artificial olfactory device based on the ion‐gel. As depicted in Figure 14e,f, recover modal and persistence modal were depended on the application of gate voltage. When *V*_G_ = 0 V and NH_3_ gas of 20 ppm were applied on the devices, the NH_3_ adsorption in the ion‐gel electrolyte influences the conductivity of channel layer, leading to a volatile memory current (Figure 14g). Conversely, upon applying a negative gate voltage, NH_3_ molecule is immersed into channel layer, thus resulting in stronger doping for higher PSC and more difficult desorption process for prolonger memory time (Figure 14h).^[^ ^168^ ^]^ These two results reveal that organic electrochemical transistors based on ion‐gel offer huge potential for the integration of nonvolatile and volatile memory in neural networks.

Another viable strategy to realize volatile memory to nonvolatile memory transition relies on the use of photons as a bridge to connect two separate devices. We recently reported a proof‐of‐concept memory optocoupler (Figure 14i) based on one synaptic light‐emitting transistor and one optical memory transistor, which can be utilized for the in‐memory display and in‐sensor computing, respectively. As illustrated in Figure 14j, the electroluminescence spectra of synaptic light‐emitting transistor span from 480 to 700 nm, which overlapped the absorption spectra of optical responsive molecules of DAEtBu in the optical memory transistor, resulting in a successful photoisomerization process triggered by synaptic light emission. We vertically stacked optical memory transistor on a synaptic light‐emitting transistor, and carried out real‐time measurements of the memory optocoupler by applying constant *V*_G1_ of 10 V, *V*_D1_ of 8 V, and *V*_D2_ of 12 V. The spiking of *V*_G2_ was implemented presynaptic stimulus, and current of *I*_DS2_ and *I*_DS1_ operated as volatile and nonvolatile PSC output, respectively. Upon weak stimulus on the synaptic light‐emitting transistor, we observed the low PSC with synaptic plasticity and negligible light emission (Figure 14k). As the training process enhanced or rehearsed, PSC increased due to conductance of channel layer increased. Upon increasing the PSC with ≈mA amplitude, excessing the built‐in threshold, long afterglow emission could be successfully activated, thus powering the optical memory transistor for a nonvolatile record, as depicted in Figure 14l. Compared with memory transition in the electronic circuit, the use of photons for the transmission of the device offers the advantages of vertical signal interaction, avoiding energy waste in conducting wires.^[^ ^169^ ^]^

## 6 Conclusions and Challenges

In summary, responsive molecules with various state toggling ability offer significant potential in the neuromorphic device fabrication. The manufacturing of an artificial brain is a multidisciplinary grand‐challenge, which can be tackled from the materials science viewpoint by exploiting stimuli responsive molecules to execute logic operations, thereby realizing neuromorphic devices implementing various algorithms to enable volatile and nonvolatile memory operations. The thermodynamics and kinetics of such interconversions can be key to fabricate in‐sensor computing devices, allowing the application in both ANNs and SNNs. The strategies for integrating of molecular switches into multiresponsive memory devices outlined in Section 4 could be expanded by using established device engineering protocols and architectures to devise unprecedented neuromorphic computing devices through the incorporation of switchable organic molecules and polymers. Despite the broad knowledge developed in this field of science which has been reported in the literature, numerous open questions and future challenges should be addressed, with the following three aspects being among the most urgent ones.

### 6.1 Versatile Responsive In‐Sensor Computing Device

Compared to the largest arsenal of molecules that are available on our planet and that has been synthesized in chemistry labs, only very few functions have been explored through their integration in working neuromorphic electronic devices. For example, magnetic responsive inorganic neuromorphic device has been demonstrated by Park and co‐workers.^[^ ^170^ ^]^ Yet, the integration of such organic molecules in magnetic responsive devices still has to be demonstrated. Also, beyond molecules responding to mechanical, light, magnetic, electrical, and redox stimuli which were discussed in this Review, other molecular systems, including those responding to temperature and pH, may be considered and embedded in neuromorphic devices.

### 6.2 Large‐Scale Fabrication of In‐Sensor Computing Device

Compared with their inorganic counterpart, the responsive organic molecules display superior design versatility, enabling their solution‐processability, thus being fully compatible with large‐scale printed fabrication technologies. Although organic neuromorphic device arrays have been demonstrated,^[^ ^171^ ^]^ several challenges must still be overcome to advance nanopattern technology and multilayer deposition. Because of the small molecular weight nature of responsive molecules, interfacing them with polymer or embedding them into cross‐linked polymer matrix may represent the effective solution for ensuring compatibility with traditional photolithography technologies. In this context, reducing the device size and decreasing the device‐to‐device variation will be a key research target in the near future.

### 6.3 Stability of Responsive Molecules and Device

In neural network computations, an accurate current read‐out is essential to reliably distinguish synaptic weights. However, the unsatisfactory stability of responsive molecules after being subjected to long‐term stimulus leads the cycle‐to‐cycle variations of devices. For example, photoswitching efficiency of device is influenced by the photo‐byproducts generation of photochromic molecules after long time exposure to a light source.^[^ ^172^ ^]^ Especially, when subjected to mechanical deformation cycles, the degeneration of functional materials also damages the stability of device. Herein, the development of robust responsive molecules with limited byproduct formation, high recovery efficiency, and high mechanical performance will be instrumental to extend the operational lifetime and enhance the reliability of devices.

## 7 Outlooks: Utilizing Memory Diversification to Realize an Artificial Brain

Upon mimicking the training‐and‐analysis toward learning as key processes ruling the human brain, all memory behavior with different retention times could be embedded into neuromorphic electronic devices. Within this context, realizing STM‐to‐LTM‐to‐PTM transition process within a single device will attract a greater interest in the research community. Moreover, harnessing the device with more sensory functionality will offer the possibility to convert various sensory stimulus signals into digital data for multimodal sensing and multisignal‐based analyses. These multifaceted efforts marked by significant advancements in material development, device fabrication, and architecture engineering, can be exploited to develop a simple prototype of artificial brain, which may empower mechanical robotic systems with various perceptions (i.e., vision, hearing, touch, taste, and smell). Toward this long‐term goal, the following advances are required.

### 7.1 Responsive Molecules with Multiple Switching States

**Figure 15:**
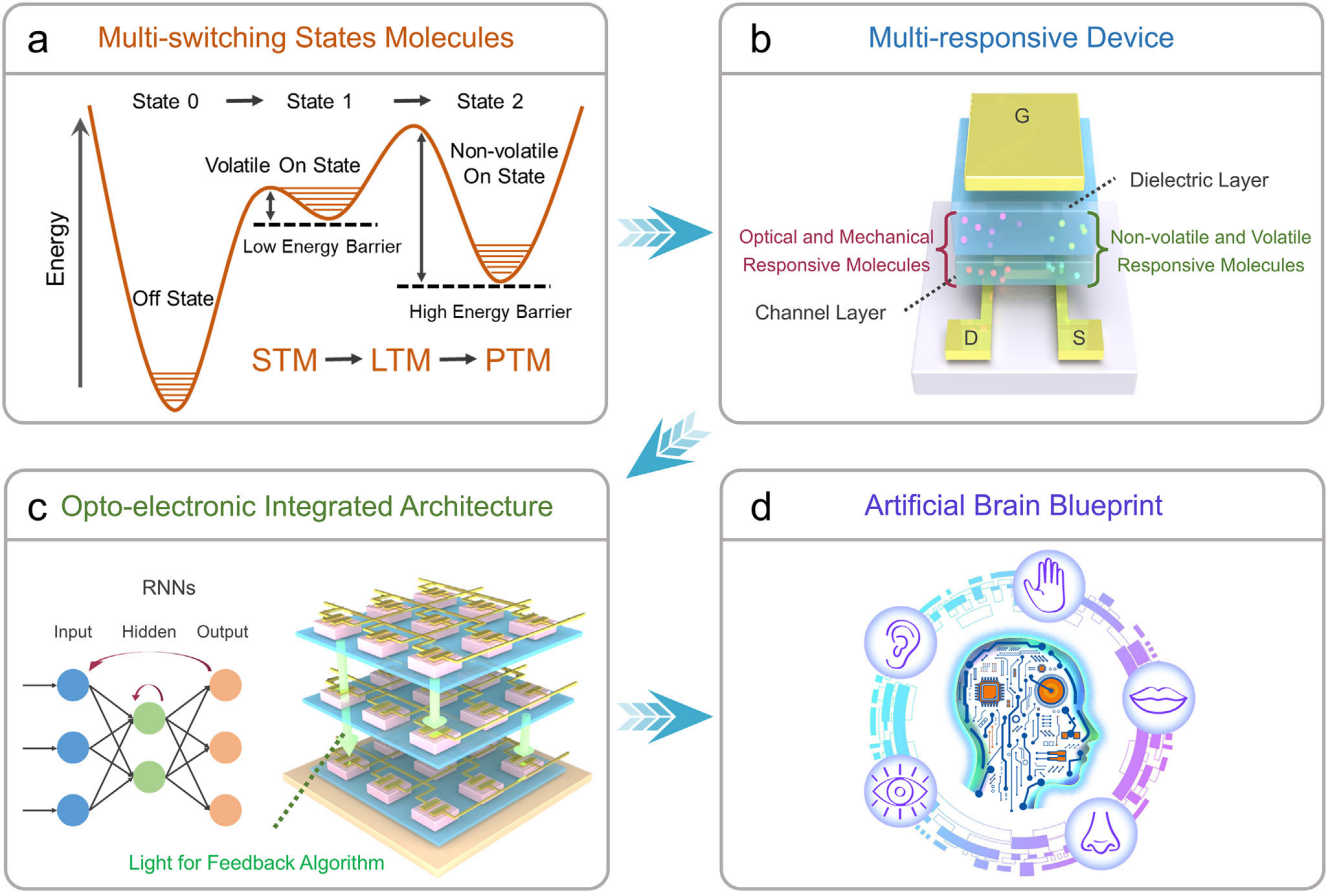
a) Energy landscape of a potential molecule displaying STM‐to‐LTM‐to‐PTM transition. b) Proposed design of a device featuring multiresponsiveness and STM‐to‐LTM‐to‐PTM transition. c) Proposed design of vertically integrated network for complex brain‐inspired logic. d) Blueprint toward artificial brain combining perception of the five senses and associated data processing.

In the AI era, the integration of logical functions to accomplish both volatile memory and nonvolatile memory is of paramount importance. Achieving STM‐to‐LTM‐to‐PTM within a single device requires responsive materials to encode multiple switching states. Some recent research efforts demonstrated that multiphotochromophoric systems, such as combined multi‐DAE, multi‐Azo photoswitches,^[^ ^173^, ^174^, ^175^ ^]^ can be synthesized to realize such hybrids. For example, Feringa and co‐workers designed a multiswitchable materials combined overcrowded alkene‐based molecular rotary motor with a DAE photoswitch.^[^ ^176^ ^]^ When the DAE is in the open form, the rotation of the motor moiety can be excited by the visible light of 455 nm, generating a thermally unstable isomerization that can be switched back by heating. Instead, upon irradiation at 312 nm the DAE interconverts to closed form and thus inhibiting the isomerization of the motor moiety, leading a thermal stable state for PTM. Such orthogonal multiphotochromic designs hold great potential for achieving memory diversification in single molecule. From the device viewpoint, a highly sought after molecular designing strategy would target at realizing the STM‐to‐LTM‐to‐PTM transition with the same light spiking. For an instance, we proposed multiswitching state molecules with thermal energy depicted in **Figure** **15**a. The single‐wavelength light can be employed for triggering two different switching processes: switching from state 0 to state 1 by initial light exposure is volatile but switching from state 1 to state 2 by prolonged light exposure is nonvolatile.

### 7.2 Multiresponsive Memory Devices

The interfacing of two or more responsive molecules with an organic or 2D semiconductor is a viable approach to construct a high‐performance FET with versatile memory responsive behavior. We recently interfaced few‐layer WSe_2_ with photochromic molecule and ferroelectric polymer (PVDF‐TrFE), thereby fabricating a multiresponsive memory device capable to respond to four independent stimuli, i.e., heat, light irradiation, and electric field stimulus simultaneously.^[^ ^177^ ^]^ In this context, toward versatile multifunctional device fabrication, a reliable solution can consist in the separate incorporation of two different responsive molecules into two different functional layers. As proposed in Figure 15b, the optical responsive molecules and mechanical responsive molecules can be embedded into the channel layer and dielectric layer, respectively. In this way, the device would be capable to accomplish object recognition task by the coperception of vison and physical touch. Furthermore, the realization of devices endowed both with nonvolatile and volatile memories can be achieved by embedding volatile and nonvolatile responsive molecules into the dielectric layer and channel layer, respectively.

### 7.3 Light as a Bridge for Active Responsive Molecule for 3D Interconnection

The 3D nature of neuron network connection in the human brain is a key characteristic which enables the transfer of information (feedback or update) between various neural layers. In some tasks aiming at the mobility pattern prediction, recurrent neural networks (RNNs) are more suitable than ANNs. In RNNs architecture, neurons in a previous layer can receive the spiking from a neuron in the next layer, enabling historical synaptic weight correction.^[^ ^178^ ^]^ From the electronic circuit fabrication viewpoint, realizing such an RNNs architecture on the neuromorphic chip requires a reliable connection between different synaptic device arrays, augmenting drastically the fabrication complexity. Vertical integration technology provides the potential to mimic this 3D connection network property, which relies on the bonding connection line between the electrodes on different layers. For example, in a typical active‐matrix light‐emitting diode (LED) structure, the connection between the top FET layer and the bottom Micro‐LED layer was achieved by the hole patterned on the isolation layer. However, this via‐hole forming method faces the limitation of device robustness, due to the low stability of functional materials during the etching or lithography processes.^[^ ^179^, ^180^ ^]^ Herein, to solve this issue, photons can be exploited for the 3D data interconnection (Figure 15c) by inserting the synaptic light‐emitting device and light‐trigged artificial synapse into the setting position of device array.^[^ ^181^, ^182^ ^]^ When the PSC of the device in the next layer reaches the threshold, light can act as a bridge to provide feedback to the previous layer, thereby fulfilling the RNNs in a simple way.

The responsiveness to different independent stimuli can be best exploited through the fabrication of multimodal sensory systems that are capable of detecting numerous independent inputs beyond the capability of human perception (Figure 15d). Such systems can be integrated into working devices for the remote and precise monitoring of the health, environment, and natural events. Overall, the integration of multiple responsive (macro)molecular systems in electronic devices can be a veritable cornerstone in neuromorphic computing, in the context of boosting the functional complexity of advanced memory devices and data processing, significantly impacting tomorrow's electronics and sensing technologies.

## Conflict of Interest

The authors declare no conflict of interest.
